# MAIT cells protect against sterile lung injury

**DOI:** 10.1016/j.celrep.2025.115275

**Published:** 2025-02-06

**Authors:** Xiawei 夏维 Zhang 张, Shuailin 帅霖 Li 李, Wojciech Lason, Maria Greco, Paul Klenerman, Timothy SC Hinks

**Affiliations:** 1 Respiratory Medicine Unit, Experimental Medicine Division, Nuffield Department of Medicine, University of Oxford, John Radcliffe Hospital, Oxford, OX3 9DU, United Kingdom.; 2 Jenner Institute, Nuffield Department of Medicine, University of Oxford, Oxford, OX3 7DQ, United Kingdom.; 3 MRC Weatherall Institute of Molecular Medicine, John Radcliffe Hospital, University of Oxford, Oxford, OX3 9DU, United Kingdom.; 4 Peter Medawar Building for Pathogen Research and Translational Gastroenterology Unit, Nuffield Department of Clinical Medicine, University of Oxford, Oxford, OX1 3SY, United Kingdom.

## Abstract

Mucosal-associated invariant T (MAIT) cells, the most abundant unconventional T cells in the lung, can exhibit a wide range of functional responses to different triggers via their TCR and/or cytokines. Their role, especially in sterile lung injury, is unknown. Using single cell RNA sequencing (scRNA-seq), spectral analysis and adoptive transfer in a bleomycin-induced sterile lung injury, we found that bleomycin activates murine pulmonary MAIT cells and is associated with a protective role against bleomycin-induced lung injury. MAIT cells drive the accumulation of type 1 conventional dendritic cells (cDC1), limiting tissue damage in a DNGR-1 dependent manner. Human scRNA-seq data revealed that MAIT cells were activated, with increased cDC populations in idiopathic pulmonary fibrosis patients. Thus, MAIT cells enhance defence against sterile lung injury by fostering cDC1-driven anti-fibrotic pathways.

## Introduction

Mucosal-associated invariant T (MAIT) cells are innate-like T cells that recognise small molecule derivatives of riboflavin synthesis ^1^ such as 5-(2-oxopropylideneamino)-6-d-ribitylaminouracil (5-OP-RU) presented on the major histocompatibility complex (MHC)-related protein-1 (MR1) ^2,3^. MAIT cells have potential for multiple diverse functions in the host response to a wide variety of bacterial, viral and fungal pathogens and in promoting tissue repair ^4–7^. MAIT cells are particularly abundant in the lung, comprising up to 10% of all pulmonary T cells in a healthy human ^8,9^. They are characterised by expression of a semi-invariant T-cell receptor (TCR)-α chain: typically Vα7.2–Jα33/12/20 in humans and Vα19–Jα33 in mice ^2,3^. These features imply an essential role of MAIT cells in pulmonary mucosal immunology, particularly during the initial stages of an immune response, yet our understanding of the full repertoire of MAIT cell functions remains incomplete, particularly in the context of tissue injury and repair. Increasing evidence implicates MAIT cells in bridging innate and adaptive immunity, an important role being the recruitment of other immune cells, particularly dendritic cells (DC) and monocytes during TCR-dependent ^10–12^ and cytokine-dependent activation ^13,14^.

Strategically located within the airway epithelium and interstitium, pulmonary DCs bridge the external and internal environments ^15,16^. The lung features two distinct conventional dendritic cell subsets (cDC, MHCII^+^ CD11c^+^): CD103^+^ type 1 cDC (CD103^+^ CD11b^lo/–^ XCR1^+^ DNGR-1^+^ SIRP-α^−^ CX3CR1^−^ F4/80^−^, cDC1) and CD11b^+^ type 2 cDC (CD11b^hi^ CD103^–^SIRP-α^+^ CX3CR1^+^ F4/80^+^, cDC2) ^17^. cDC1 specialize in cross-presenting antigens to CD8^+^ T cells, promoting Th1 cells. In contrast, cDC2 excel at stimulating CD4^+^ T cell responses, mainly Th2 or Th17 cells. Lung cDCs originate from common dendritic cell precursors (CDPs) in the bone marrow. These CDPs mature into pre-dendritic cells (pre-DCs), which migrate to the lungs through the bloodstream and differentiate into either cDC1 or cDC2 subsets guided by local signals and specific transcription factors ^18–20^.

DCs play a regulatory role in pulmonary fibrosis, accumulating in the lungs in idiopathic pulmonary fibrosis (IPF) patients ^21–23^ and in bleomycin mouse models ^24,25^, whilst diminishing in the circulation ^26^. Pulmonary cDC1s increase with bleomycin treatment but are reduced with transforming growth factor (TGF)-β inhibition, suggesting anti-inflammatory and anti-fibrotic roles in pulmonary fibrosis ^25^. Moreover, increased fibrosis severity and impaired lung function were seen in DC-deficient mice, but mitigated when DC counts were boosted ^27^, though their protective mechanism is still unclear.

In this study, we aim for the first time to define the role of MAIT cells in sterile lung injury and to investigate the underlying mechanisms. We employed a model of lung injury using bleomycin: a potent chemotherapeutic agent, with a well-characterised side effect profile of acute lung injury, followed by a chronic phase with pathological hallmarks of human IPF ^28^. We have shown for the first-time that MAIT cells accumulate and are activated upon sterile injury, in a cytokine-dependent manner, and we have discovered an *in vivo* mechanism by which pulmonary MAIT cells make an important contribution to protection against sterile lung tissue damage in mice.

## Results

### Activated MAIT cells accumulate in the lung upon bleomycin treatment in a cytokine-dependent manner

**Fig. 1:**
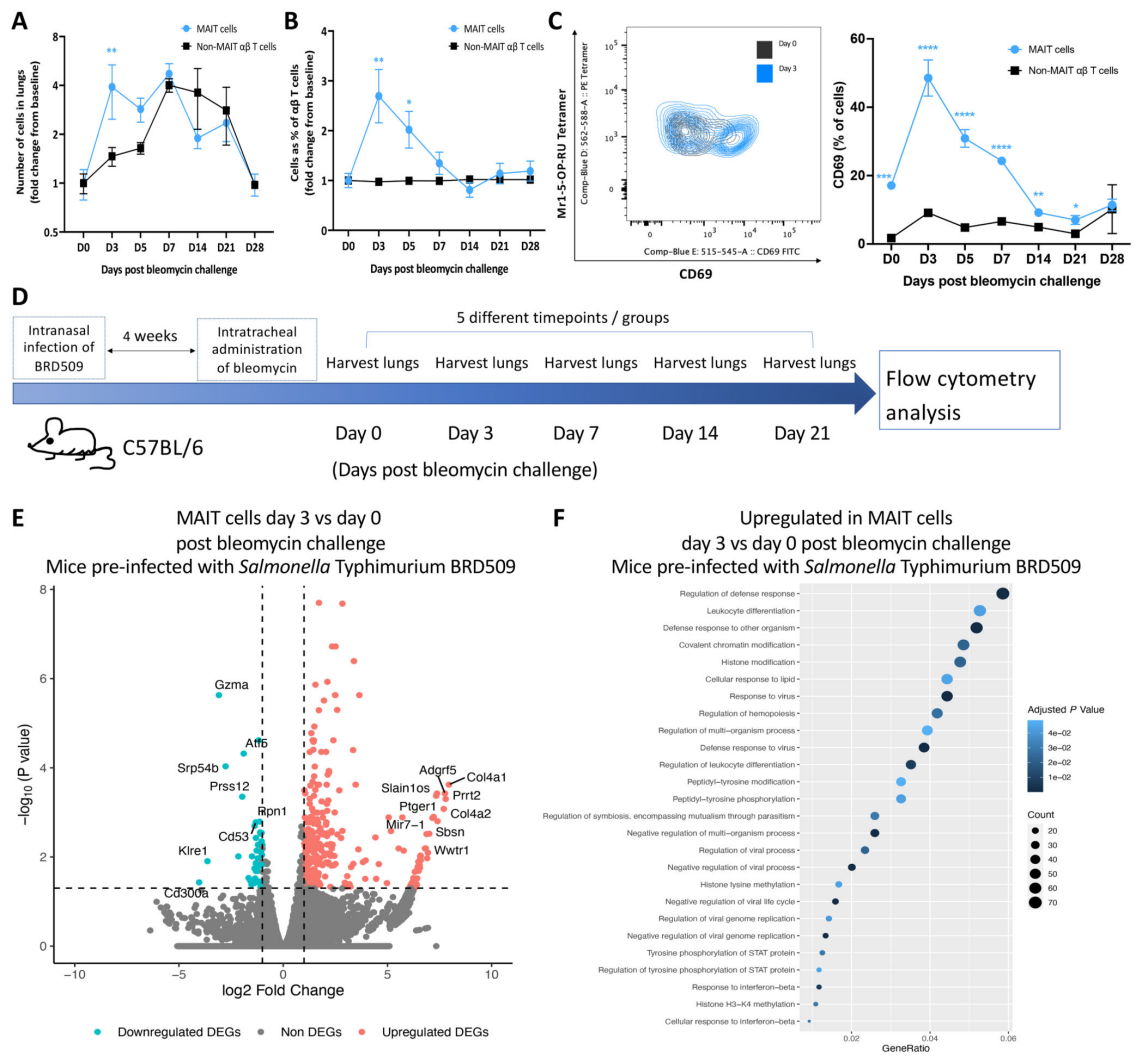
Bleomycin induces accumulation and activation of pulmonary MAIT cells. Experiments used mice without *Salmonella* Typhimurium pre-infection (**A**-**C**) or mice previously infected with *Salmonella* Typhimurium BRD509 (**E**-**H**). (**A**) Fold change in absolute pulmonary MAIT and non-MAIT αβT cells post-bleomycin challenge, relative to PBS controls (D0). MAIT and non-MAIT αβ T cells compared with unpaired *t* or Mann-Whitney tests. (**B**) Fold change in pulmonary MAIT and non-MAIT αβ T cell frequency as percentage of total pulmonary αβ T cells *versus* unchallenged controls. MAIT and non-MAIT αβ T cells compared with unpaired *t* or Mann-Whitney tests. (**C**) CD69 expression in naïve mice, comparing between MAIT and non-MAIT αβ T cells at individual time-points using either unpaired *t* or Mann-Whitney tests. (**A**-**C**) The data are presented as the mean ± SEM of a single experiment, with 3-5 mice in each group. (**D**) Protocol schematic. (**E**) DEGs [log2 fold change (FC)>1, adjusted *P*<0.05] of pulmonary MAIT cells day 3 post-bleomycin challenge *versus* PBS controls (n=3/group). Top up/down-regulated genes annotated. Horizontal line *P=*0.05; Vertical line log2 fold change=1. (**F**) Top 25 enriched (*P*<0.05) Gene Ontology (GO) (biological process) pathways among upregulated DEGs day 3 post-bleomycin *versus* PBS controls. Colour intensity indicates statistical significance; dot size represents number of genes upregulated per pathway; x-axis shows proportion of all DEGs included in pathway (Gene Ratio).

Our initial objective was to establish whether sterile lung injury could stimulate pulmonary MAIT cells *in vivo*. We administered bleomycin intratracheally to C57BL/6 (wild type, WT) mice, to precipitate acute, sterile lung inflammation, followed by a tissue repair phase and subsequent fibrosis over a fortnight ^28^. We detected an earlier surge of pulmonary MAIT cells (characterised as CD45.2^+^ TCRβ^+^ CD19^−^ MR1-5-OP-RU tetramer^+^ cells, fig. S1A) compared to non-MAIT αβ T cells, as illustrated by the fold change in their absolute number 3 days post-bleomycin challenge over baseline (Fig. 1A and fig. S2, G and J). On both day 3 and 5 post-challenge, the fold change in the proportion of total αβ T cells was significantly higher in MAIT cells than that in non-MAIT αβ T cells (Fig. 1B and fig. S2, H and K). Moreover, pulmonary MAIT cell CD69 expression increased significantly at days 3 and 5 post challenge relative to unchallenged controls, and CD69 expression was significantly higher in MAIT cells than that in non-MAIT αβ T cells (Fig. 1C, fig. S2, I and L).

In nature, early life microbial exposures are essential for the development of MAIT cell populations. When mice have been raised in a specific pathogen free environment, MAIT cells constitute less than 1% of total pulmonary αβ T cells ^29^ but can be up to 10% in healthy humans ^8,9^. Therefore, we employed an established MAIT cell-enriched model by infecting WT C57BL/6 mice intranasally with 10^6^ CFU *Salmonella* Typhimurium BRD509 four weeks prior to initial bleomycin challenge for clearer population delineation (Fig. 1D and fig. S1, B to D) ^30,31^. This bacterial inoculum is rapidly cleared from the lungs but transiently provides the required combination of MAIT cell ligands and pathogen-associated molecular patterns necessary to produce rapid and lasting expansion of the MAIT cell population ^30,32^. Transcriptomic analysis of MAIT cells isolated from mice with and without *Salmonella* Typhimurium BRD509 infection revealed that infected mice exhibited upregulation of both Type 1 and Type 17 immune signature genes in their MAIT cells. These genes include *Ifng, Tnf, Bhlhe40*, and *Ccr5* for Type 1, and *Il17a, Rorc, Il1r1*, and *Lamc1* for Type 17 (fig. S1F). This is consistent with the expanded hybrid MAIT1/MAIT17 cells found in the lung post pulmonary infection with *Salmonella* Typhimurium BRD509 ^30,31,33^ or *Francisella tularensis* ^34^ and a hybrid MAIT1/MAIT17 phenotype commonly seen in human MAIT cells ^35^, which is absent in naïve mice ^36^.

While it was previously reported that post-*Salmonella* Typhimurium BRD509 infection MAIT cells adopt an effector memory phenotype, with relatively high baseline CD69 expression (fig. S1E) ^30^, we still observed rapid MAIT cell accumulation, peaking on day 3 post-challenge (fig. S2, A and B, M and N), along with activation upon bleomycin stimulation, evidenced by significantly increased CD69 expression on day 3 and 7, compared to unchallenged PBS controls (fig. S2, C and O). Conversely, no significant changes were observed in either the accumulation or activation of non-MAIT αβ T cells post-bleomycin challenge (fig. S2, A and B, P to R). Collectively, these results indicate MAIT cells accumulate and are activated early in the lungs following bleomycin-induced sterile injury of mice.

As MAIT cells can be activated in a TCR-independent manner by cytokines, including interleukin (IL)-12, -15, -18, and type I interferon (IFN), in antiviral responses ^37^, we examined MAIT cell responses post bleomycin challenge utilising mouse strains deficient in the two most important of these pathways, IL-18R or IFN-αR without a preliminary MAIT boost. Relative to WT C57BL/6 mice, we observed a marked impairment in pulmonary MAIT cell accumulation on day 3 post-bleomycin in both IL-18R and IFNαR-deficient mice (fig. S2, D and E, S and T, V and W). Similarly, MAIT cell activation was significantly impeded in the absence of IFNαR or IL-18R (fig. S2, F, U and X). These findings suggest bleomycin-induced MAIT cell activation is predominantly cytokine driven, with IFN-αR and IL-18R playing key roles.

Next, we assessed the transcriptomic consequences of bleomycin induced MAIT cell activation in lungs of *Salmonella* Typhimurium-treated mice using bulk RNA-seq of flow-sorted pulmonary MAIT cells. The numbers of DEGs in bleomycin-challenged lung MAIT cells compared with unchallenged controls, were 425 (361 up, 64 down), 1230 (399 up, 831 down), 0, 24 (4 up, 20 down) and 131 (40 up, 91 down) genes at day 3, 7, 14, 21 and 28 post-bleomycin challenge, respectively (fig. S3A, for full list of DEGs see data S1). Intriguingly, the top 15 upregulated genes in pulmonary MAIT cells at day 3 post-bleomycin included tissue-damage related genes such as *Col4α1, Col4α2, Ptger1*, and *Wwtr1* (Fig. 1E). The predominant GO pathways upregulated in MAIT cells at day 3 post-bleomycin challenge compared to those from unchallenged mice were associated with the regulation of the defence response, leukocyte differentiation, and response to viruses (Fig. 1F). We also observed a notable rise in the *Cd69* gene expression and a modest upregulation of several inflammatory cytokines, such as *Csf2, Ifng, Tnf, Il17a, Il10* and *Il22*, in MAIT cells (Fig. S3B), and verified selected cytokines by flow cytometry (fig. S3C and S3D).

To ascertain whether pre-infection with *Salmonella* Typhimurium BRD509 significantly altered the transcriptome of MAIT cells, we conducted bulk RNA sequencing using naïve MAIT cells at earlier timepoints post-bleomycin challenge, without pre-infection of *Salmonella* Typhimurium BRD509. We identified 544 and 339 differentially regulated genes in lung MAIT cells at day 3 and day 7 post-bleomycin challenge, respectively (fig. S4A, data S2). The top predominant GO pathways upregulated in MAIT cells at day 3 post-bleomycin challenge, in comparison to those from unchallenged mice, were associated with the response to viruses, regulation of the inflammatory response, regulation of cytokine-mediated signalling pathways, and regulation of type I interferon production (fig. S4B). To evaluate the consistency of DEGs in MAIT cells, both with and without pre-infection, we conducted a GO enrichment analysis on the DEGs shared by MAIT cells under both conditions (Day 3 post-bleomycin versus PBS control). The predominant GO pathways of shared upregulated genes in both conditions were the response to virus, positive regulation of the innate immune response, regulation of cytokine-mediated signalling pathways, and regulation of type I interferon production (fig. S4C), which are similar to the enriched GO terms in naïve MAIT cells.

### MAIT cell-deficient mice show dysregulated pulmonary immune responses upon bleomycin challenge

**Fig. 2:**
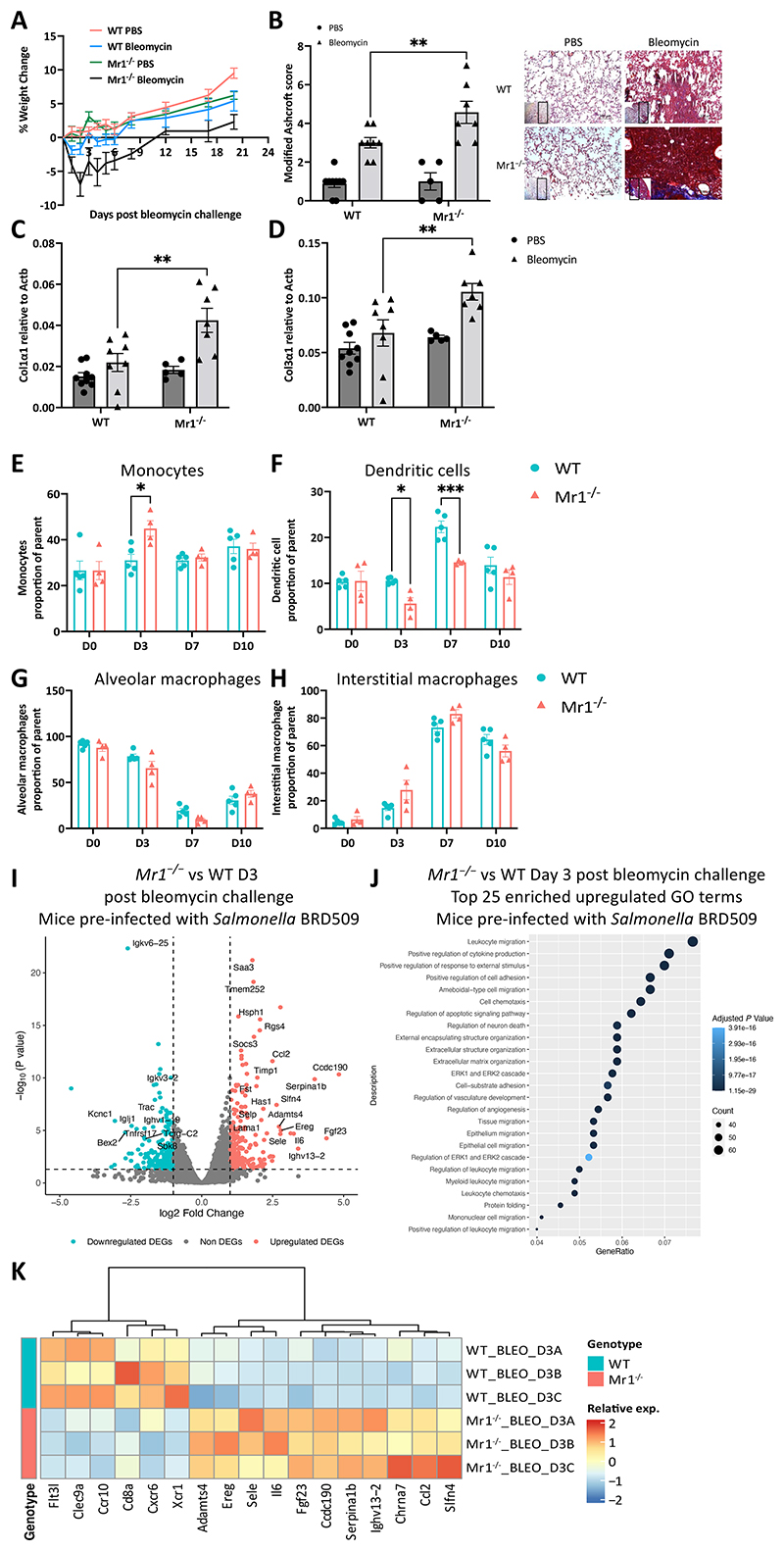
Dysregulated immune responses in the lungs of MAIT cell-deficient mice following bleomycin challenge. (**A** to **K**) Experiments used mice pre-infected with *Salmonella* Typhimurium BRD509. (**A**) Body weight loss shown as a percentage from before the bleomycin challenge. (**B** and **C**) Modified Ashcroft score (**B**) and representative Masson’s trichrome-stained lung slices (**C**) of PBS or bleomycin-challenged WT and *Mr1*^*−/*−^ mice at day 21. (**D**) Gene expression levels of *Col1α1*, and *Col3α1* in lung homogenates of PBS or bleomycin-challenged WT and *Mr1*^−/−^ mice at day 21. *Actb* was used as a housekeeping gene. (**E** to **H**) Frequencies of monocytes (**E**), dendritic cells (**F**), alveolar macrophages (**G**) and interstitial macrophages (**H**) as percentages of parent in lungs post-challenge. (**A** to **H**) Data are one representative experiment of two independent experiments, with 4–6 mice per group in each replicate. Graphs show mean ± SEM. Statistical significance tested by two-way ANOVA with Holm-Sidak’s multiple comparisons test; *P < 0.05, **P < 0.01, ***P < 0.001. (**I**) Volcano plot of DEGs [log2 fold change (FC) > 1, adjusted *P* < 0.05] in whole lung tissue between *Mr1*^−/−^ and WT mice lungs at day 3 post-bleomycin challenge. The top up and down-regulated genes are labelled. Horizontal and vertical lines indicate *P* value and log2 FC thresholds of 0.05 and 1, respectively. (**J**) Top 25 significantly enriched (*P* < 0.05) pathways from GO database (biological process) in upregulated DEGs in the lungs of *Mr1*^−/−^ mice compared with WT mice lungs at day 3 post-challenge (n=3/group). Colour intensity shows the statistical significance of the enrichment and dot size shows the number of genes upregulated in the pathway. The x axis shows the proportion of all DEGs included in the pathway (Gene Ratio). (**K**) Heatmap showing relative expression of selected genes of mice lungs at day 3 post bleomycin comparing WT to *Mr1*^−/−^ mice.

We next sought to determine whether the recruitment and activation of MAIT cells in response to bleomycin have an impact on the phenotype. To this end, we assessed weight loss and tissue damage between WT and MAIT cell-deficient *Mr1*^−/−^ mice. Both WT and *Mr1*^−/−^ mice were subjected to 10^6^ CFU *Salmonella* Typhimurium BRD509 infection to expand the MAIT cell population followed by intratracheal bleomycin administration four weeks post-MAIT cell enrichment (Fig. 1D). Notably, *Mr1*^*−/−*^ mice exhibited more substantial weight loss (Fig. 2A), heightened tissue damage (Fig. 2B), and increased gene expression of *Col1α1* and *Col3α1* compared to WT mice (Fig. 2, C and D). Hydroxyproline levels in the lungs showed no significant difference between WT and *Mr1*^−/−^ mice (fig. S5A).

To assess whether pre-infection with *Salmonella* Typhimurium BRD509 is necessary for the protective effects of MAIT cells, we evaluated weight loss in WT and *Mr1*^*−/−*^ mice, without prior *Salmonella* Typhimurium BRD509 infection. Remarkably, even in the absence of pre-infection, *Mr1*^−/−^ mice experienced significantly greater weight loss compared to WT mice (fig. S5B). This indicates that the protective role of MAIT cells does not rely on prior infection with *Salmonella* Typhimurium BRD509.

For the bleomycin challenge, *Salmonella* Typhimurium BRD509 pre-infected mice received 1.875 U/kg, while naïve mice were treated with 1.0 U/kg. The higher dose in pre-infected mice was necessary due to their reduced susceptibility to bleomycin-induced weight loss. Naïve mice treated with a sublethal, lower dose exhibited more significant weight loss compared to mice pre-infected with *Salmonella* Typhimurium BRD509 (fig. S5C), indicating greater vulnerability. It has been suggested that the protective role of MAIT cells is more pronounced under conditions of high disease burden or compromised immunity, reflecting the potential masking effect of the role of MAIT cells in naïve models ^38^. The sublethal dose in naïve mice is difficult to control due to variability in baseline weight, which can lead to less reproducibility of results. In contrast, the MAIT cell-boosted mouse model does not require a sublethal dose, making it more robust for inducing differences between WT and *Mr1*^*−/−*^ mice. Furthermore, this model more accurately reflects the MAIT cell frequencies observed in human lungs, where these cells account for up to 10% of the total T cell population (fig. S1D). Prior *Salmonella* Typhimurium infection allows MAIT cells to more closely resemble the phenotypic characteristics of human MAIT cells (fig. S1F), prompting us to continue using this pre-infection model in subsequent experiments. This model, therefore, provides a setting that better mirrors human lung conditions, enabling us to investigate the protective effects of MAIT cells in the context of sterile lung injury.

To elucidate the mechanism underlying the observed phenotypic differences, we evaluated immune cell infiltration between WT and *Mr1*^−/−^ mouse lungs using spectral flow cytometry analysis (Cytek Aurora) following a published gating strategy ^39^ (fig. S6A). Lung samples were collected on days 0, 3, 7 and 10 post challenge (Fig. 1D). *Mr1*^−/−^ mice exhibited a decreased frequency of DCs (MerTK^-^ CD11c^+^ MHCII^+^) on days 3 and 7, and an increased frequency of monocytes on day 3 post bleomycin challenge. Notably, differences in the frequencies of alveolar macrophages, interstitial macrophages, neutrophils, eosinophils, NK cells, NK T cells, CD4^+^ T cells, CD8^+^ T cells, and γδ-T cells post-challenge between WT and *Mr1*^−/−^ mice were not statistically significant (Fig. 2, E to H; fig. S6, B to U).

We then investigated the transcriptomic variations between WT and *Mr1*^−/−^ mouse lungs. Accordingly, we obtained bulk RNA-seq data from whole mouse lungs pre-infected with BRD509 and challenged with bleomycin. Lung samples were collected on days 0, 3, 7, 14 and 21 post challenge (Fig. 1D). Compared with WT mice, 9 (3 up, 6 down), 1729 (967 up, 762 down), 116 (54 up, 62 down), 173 (65 up, 108 down) and 192 (107 up, 85 down) genes were differentially expressed in *Mr1*^−/−^ mice lungs at days 0, 3, 7, 14 and 21 post-bleomycin challenge, respectively (fig. S6V, Fig. 2I, and data S3).

The chemokine *Ccl2* (monocyte chemoattractant protein-1, MCP-1), which recruits myeloid cells towards sites of inflammation, and the proinflammatory cytokine *Il6* were prominently upregulated in *Mr1*^−/−^ mice at day 3. Both CCL2 and IL-6 are known contributors to lung fibrosis ^40^. Gene Ontology (GO) enrichment analysis for biological processes ^41^ revealed only 3 significantly enriched (*P* < 0.05) upregulated gene sets in *Mr1*^−/−^ mice lungs versus WT mice lungs without bleomycin challenge (fig. S6W), but 1526 significantly enriched gene sets at day 3 post-bleomycin, of which the top ranked terms include leukocyte migration, regulation of cytokine production, regulation of response to external stimulus, cell adhesion, migration and chemotaxis (Fig. 2J).

Notably, expression of *Xcr1, Clec9a*, markers of DC ^42^ – and *Flt3l* and *Ccr10*, both essential for DC development and recruitment ^42,43^, were significantly downregulated in *Mr1*^−/−^ mice relative to WT mice at day 3 post-bleomycin (Fig. 2K), consistent with downregulation of the DC population in *Mr1*^−/−^ mice relative to WT after bleomycin challenge shown in flow cytometry analysis. Our data imply that MAIT cells may modulate the accumulation of immune cells in the lung after sterile lung challenge, notably DCs, during sterile lung challenges.

### MAIT cells protect against bleomycin-induced sterile lung injury via cDC1-DNGR-1 signalling pathway

**Fig. 3:**
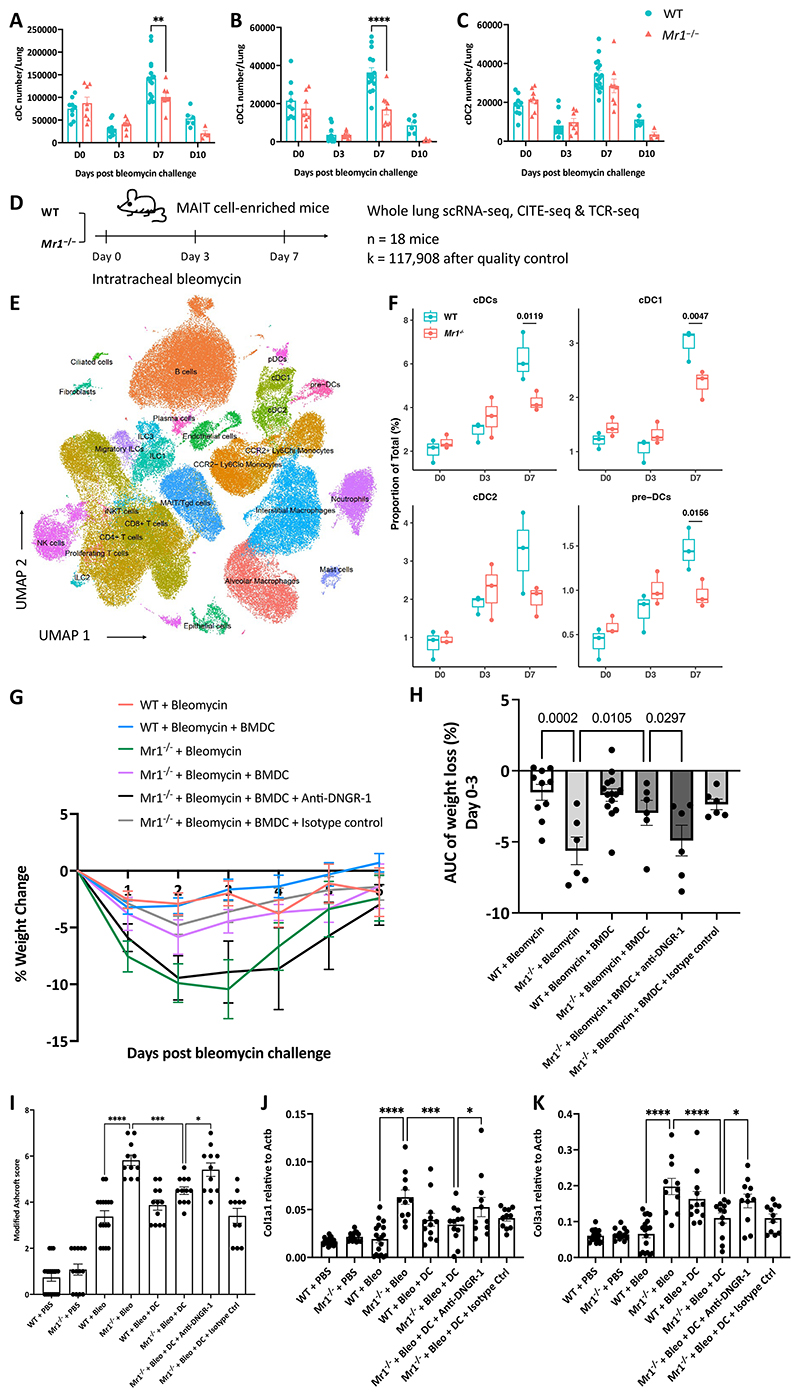
MAIT cells protect against bleomycin-induced sterile lung injury via cDC1-DNGR-1 signalling pathway. (**A** to **C**) Absolute numbers of total cDCs (**A**), cDC1 (**B**) and cDC2 (**C**) in WT and *Mr1*^−/−^ mice lungs post-bleomycin challenge. Data (mean ± SEM) represent combined results from two independent experiments conducted on Days 0, 3, and 7, and one experiment on Day 10, with each group consisting of 3-6 mice. (**D**) Single cell suspensions from whole-mouse lungs were analysed using scRNA-seq at the indicated time points after bleomycin-mediated lung injury. (**E**) UMAP embedding of 117,908 high-quality single cells colour-coded by predicted cell lineage. (**F**) Proportion of the indicated cell types of total lung cells was calculated for individual mice at the indicated time points at baseline (PBS control, Day 0) and after bleomycin challenge (Day 3 and 7) (n = 3 for each genotype). *P* values generated using a two-way ANOVA with Sidak’s multiple comparisons test. (**G**) Body weight loss expressed as a percentage of the weight before bleomycin challenge. Adoptive transfer, performed twice, used 5 × 10^5^ BMDCs from mice infected with *Salmonella* Typhimurium BRD509 on day 28 post-infection, maintaining consistent baseline conditions with the recipient mice. BMDCs were transferred 1-day post-bleomycin challenge. (**H**) Body weight loss as area under the curve (AUC). (**I**) Modified Ashcroft score of lung slices of PBS or bleomycin-challenged WT and *Mr1*^−/−^ mice at day 21, stained with Masson’s trichrome. (**J** and **K**) Gene expression of *Col1α1* (**J**), and *Col3α1* (**K**) in lung homogenates of PBS or bleomycin-challenged WT and *Mr1*^−/−^ mice at day 21. *Actb* was used as a housekeeping gene. For weight loss results, data are one representative experiment of two independent experiments, with 4–6 mice per group in each replicate. For histology score and RT-qPCR results, data were pooled from two independent experiments (n=4-6 per group). Graphs show mean ± SEM. Statistical significance tested by one-way ANOVA with Holm-Sidak’s multiple comparisons test; *P < 0.05, **P < 0.01, ***P < 0.001, ****P < 0.0001.

We subsequently aimed to explore the subpopulations of DCs to determine which subsets contributed to the decreased accumulation observed in *Mr1*^−/−^ mice, following established guidelines ^44^ (fig. S7A). We noted an accumulation of CD45^+^ CD64^-^ CD11c^+^ MHCII^+^ cDCs (Fig. 3A and S7B), particularly CD103^+^ cDC1, in lungs of WT mice on day 7 post-bleomycin. In contrast, *Mr1*^−/−^ mice failed to accumulate CD103^+^ DCs, with significantly lower total count and percentage of pulmonary CD103^+^ DCs at day 7 post-challenge compared to WT counterparts (Fig. 3B and S7C). There was also a tendency towards impaired CD11b^+^ cDC2 accumulation in *Mr1*^−/−^ lungs on day 7 post-bleomycin but this did not reach statistical significance (Fig. 3C and S7D).

To assess functional differences in cDC1 we investigated surface expression of activation markers (fig. S7E). No discernible difference in the expression of co-stimulatory molecules CD86 (Fig. S6F) or CD40 (fig. S7G) was observed in the first week. Additionally, we detected no variation in the levels of CCR2, known to facilitate DC migration and recruitment ^45^, or DNGR1, a C-type lectin receptor exclusively expressed in cDC1, which is encoded by Clec9a ^46^, on cDC1 between WT and *Mr1*^−/−^ mice lungs at day 7 post-bleomycin (fig. S7H and I). However, on day 10 post bleomycin-induced lung damage, we did see an upregulation of CD40 and CCR2 in lung cDC1 cells from *Mr1*^−/−^ mice, suggesting that the cDC1s are showing a delayed inflammatory response in the *Mr1*^−/−^ mice.

To comprehensively delineate the cellular dynamics of major cell lineages post-bleomycin injury, we utilised single-cell RNA sequencing (scRNA-seq) (10x Genomics), Cellular Indexing of Transcriptomes and Epitopes by Sequencing (CITE-seq) and TCR sequencing. We again used the previously described MAIT-cell enriched mouse model, and obtained single cell suspensions from both WT and *Mr1*^−/−^ whole lungs of PBS-treated controls, as well as at days 3 and 7 post-injury, with three replicates for each time point (Fig. 3D). We obtained transcriptomes from 117,908 cells following quality control filtering. Principal component analysis highlighted variability influenced by both timepoints and mouse genotype (fig. S8A and B). Post-data integration and unsupervised clustering analysis, 27 cell type identities were annotated using canonical marker genes and existing scRNA-seq datasets from mouse lungs ^47–49^ (Fig. 3E, fig. S8C). All lineages were observed across both WT and *Mr1*^−/−^ mice at all three timepoints (fig. S8, D and E). MAIT cells exhibited a single-cell transcriptional profile akin to γδ-T cells, leading to a shared cluster in the Uniform Manifold Approximation and Projection (UMAP) (Fig. 3E). Expectedly, MAIT cells were absent in *Mr1*^−/−^ mice (fig. S8F), but there was a non-significant tendency towards an increase in CD4^+^ and CD8^+^ T cells in *Mr1*^−/−^ mice compared to WT mice (fig. S9), possibly due to a compensatory mechanism.

We observed an accumulation of cDC1 (*P*=0.0047) and cDC2 (non-significant) in the lungs of WT mice but not in *Mr1*^−/−^ mice. These observations align with the flow cytometry data depicting a deficiency in cDC1 accumulation in *Mr1*^−/−^ mice (Fig. 3F). Concomitantly, accumulation of monocyte and NK cells was prominently detected in the lungs of *Mr1*^−/−^ mice, whereas this was not the case in WT counterparts (fig. S9). Furthermore, in the lungs of WT mice, there was a discernible expansion of both interstitial macrophages and fibroblasts (fig. S9), and such expansions were absent in the *Mr1*^−/−^ mice.

Next we investigated the differences in cell type-specific DEGs between *Mr1*^−/−^ and WT mice (fig. S10 and data S4). Gene expression differences across most cell types were limited, with very few DEGs identified – fewer than 64 in any given cell type. Amongst pulmonary DCs and monocytes, we observed downregulation of genes in *Mr1*^−/−^ mice on both day 3 and 7 post-bleomycin, including *Pbx1, Fcgr2b* and *Sh2d1b*1 (fig. S10, data S4). The inhibitory Fc receptor *Fcgr2b* was consistently downregulated in *Mr1*^−/−^ cDC1 across all time points. This is paired with downregulation of *Cxcl1*/*Cxcl2* in cDC2 on day 7 post-challenge (fig. S10), suggesting a disrupted chemokine expression profile within the cDC2 of *Mr1*^−/−^ mice.

We then looked at the precursor cells leading to cDCs – pre-DC population, in the lungs of both mice strains. Derived from CDP in the bone marrow, pre-DCs traffic to various tissues where they differentiate into cDC1 or cDC2, contingent upon tissue-specific and local environmental cues ^18–20^. Employing Monocle2 for trajectory analysis ^50^, we discerned a clear differentiation pathway commencing from pre-DCs (CD45^+^ MHCII^+^ CD11c^−^ Flt3^hi^ SIRP-α^−^) and culminating in cDC1 and cDC2 subsets (fig. S11A). During the differentiation from pre-DCs into cDC1 and cDC2, we noticed that *Irf8* levels go up in cDC1 but go down in cDC2. Conversely, *Irf4* levels rise in cDC2 and fall in cDC1 (fig. S11B). Of particular interest, *Mr1*^−/−^ mice exhibited a diminished pre-DC population (Fig. 3F). However, when contrasting the transcriptomic profile of pre-DCs from WT and *Mr1*^−/−^ mice, differential gene expression was minimal (fig. S11C). This suggests that, during the bleomycin challenge, MAIT cells predominantly modulate the accumulation dynamics of the pre-DC population, without substantially altering their functional profile.

To test enrichment of pathways we performed GSEA analysis for all cell types across timepoints (fig. S12). A proinflammatory response was upregulated across various cell types in *Mr1*^−/−^ mice compared with WT following bleomycin. Notably, NK cells, ciliated cells, and endothelial cells exhibited this exaggerated response on day 3, while monocytes and interstitial macrophages demonstrated a similar response on day 7, which would be expected to contribute to enhanced systemic inflammation. In summary, MAIT cells predominantly influence accumulation of immune cells in response to sterile lung injury, particularly by increasing the number of cDC1s, pre-DCs and interstitial macrophages, but have more limited influence on the transcriptome of most cell types.

We reported compromised accumulation of cDC1 in *Mr1*^−/−^ mice in the absence of MAIT cells. Given the exclusive presence of DNGR-1, a C-type lectin receptor, in cDC1 and its crucial role in managing tissue damage by detecting actin filaments exposed on necrotic cell death ^51^, we further probed its specific influence in our context. Previous studies have shown that DNGR-1 in DCs limits tissue damage in pancreatitis by dampening neutrophil recruitment, and DNGR-1 also controls neutrophil recruitment and pathology associated with systemic candidiasis ^52^. We therefore examined the role of cDC1-DNGR-1 in weight loss and tissue fibrosis in both WT and *Mr1*^−/−^ mice following bleomycin challenge. Consistent with previous data (Fig. 2, A to D), *Mr1*^−/−^ mice showed greater weight loss (Fig. 3G), more pronounced tissue fibrosis (Fig. 3H and fig. S13A), and elevated gene expression of *Col1α1* and *Col3α1* on D21 post-bleomycin challenge, when in comparison with WT mice (Fig. 3I and J). Significantly, weight loss and tissue fibrosis in *Mr1*^−/−^ mice was alleviated by intranasal adoptive transfer of Flt3L-generated bone marrow-derived dendritic cells (FLT3L-BMDC) (fig. S13, B to D) on day 1 post-bleomycin challenge (Fig. 3G-J). This alleviating effect was abrogated by antibody blockade of DNGR-1 but persisted in *Mr1*^−/−^ mice treated with isotype control (Fig. 3G-J). These results suggest that the protective effects offered by MAIT cells are mediated, at least partially, by regulating cDC1, and that cDC1s curb tissue damage through DNGR-1 signalling.

### Human scRNA-sequencing datasets demonstrate differences in MAIT cell and cDC population in IPF versus non-fibrotic control lungs tissues

**Fig. 4:**
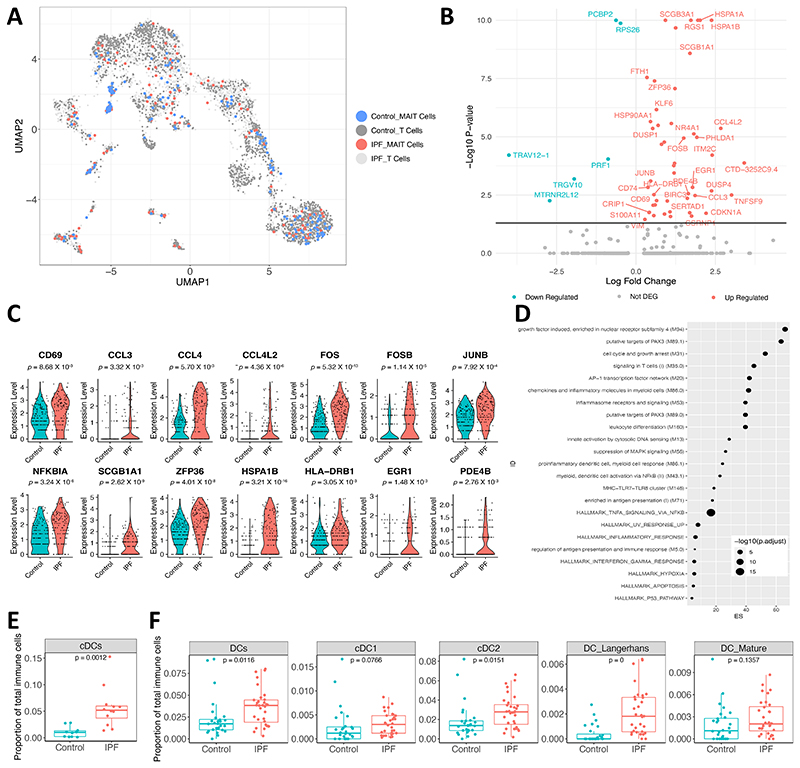
Differential gene expression and cellular frequencies in IPF patient lung MAIT cells. (**A**) UMAP showing the MAIT cells and other T cells after reintegration. (**B**) Volcano plot displays DEGs (adjusted *P* < 0.05) in IPF lung MAIT cells relative to controls. The 25 most upregulated and downregulated genes are annotated. (**C**) Violin plots illustrate expression levels of specified genes in MAIT cells, contrasting IPF with controls (GSE135893). (**D**) Analysis of DEGs for overrepresentation of blood transcriptional modules (BTM). The top 25 pathways significantly enriched (*P* < 0.05) from BTM are shown, contrasting IPF lung MAIT cells with controls. Dot size corresponds to the adjusted *P* value for each pathway, while the x-axis depicts the enrichment score (ES), calculated as the ratio of gene ratio to background ratio. (**E**) Boxplots showing the proportion of cDC relative to total immune cells in lungs of IPF patients versus controls using data from GSE135893. (**F**) Boxplots present frequencies of various DC subsets, including cDC1, cDC2, Langerhans, and mature DC, as proportions of total immune cells in lungs of IPF patients and controls, sourced from GSE136831.

To compare our murine findings with human clinical data from pulmonary fibrosis, we examined published scRNA-seq datasets of IPF in the Human Lung Cell Atlas (HLCA) ^53^ and the IPF Cell Atlas^54^. MAIT cells were prominently identified only in one dataset ^55^, where 176 MAIT cells were identified in non-fibrotic controls and 141 in IPF patients (GEO reference: GSE135893) (Fig. 4A). Two datasets ^55,56^ provided adequate participant numbers to compare cell proportions between IPF and controls (GEO reference: GSE135893 and GSE136831).

MAIT cells from IPF patients’ lungs (n=10) exhibited 55 DEGs (49 up, 6 down, Table S1) compared to controls (n=6). Notably, the MAIT cell activation marker, CD69, was among the prominently upregulated genes (Fig. 4B and C). We also observed a significant upregulation of chemokines in MAIT cells, including *CCL3, CCL4*, and *CCL4L2*, which play a pivotal role in the recruitment and activation of immune cells. Additionally, MAIT cells demonstrated enhanced expression of *FOS, FOSB*, and *JUNB* – integral components of the AP-1 transcription factor complex, which might indicate alterations in cellular signalling and responses to lung injury. The elevated expression of *NFKBIA*, an inhibitor of the NF-kB transcription factor, suggests potential modulations in inflammatory response pathways. And the elevated anti-inflammatory genes *SCGB1A1*^57^ and *ZFP36*^58^ in MAIT cells suggesting their modulatory role and a potential protective mechanism in the lungs of IPF patients.

We conducted an overrepresentation analysis of these DEGs using blood transcriptional modules (BTMs) ^59^. This analysis pinpointed a pronounced activation of the AP-1 transcription factor network, chemokines and inflammatory molecules in myeloid cells, as well as heightened activity related to pro-inflammatory dendritic cells and myeloid cell responses in IPF (Fig. 4D).

In the GSE135893 dataset, cDC (marked by the genes: *FCER1A, CD1C*, and *CLEC9A*) emerged as the sole cell population with a significant increase in IPF patients’ lungs (Fig. 4E and fig. S14B). Based on gene expression, these cDCs express *CD1C, PKIB*, and *CLEC10A*, suggesting that they are phenotypically cDC2 (fig. S14A). Analysis of the GSE136831 dataset revealed an elevation in the DC population within IPF patients’ lungs, with significant accumulations specifically in cDC2 (marked by *FCGR2B, CLEC10A, FOXN3, ABHD12*) and Langerhans cells (indicated by *CD1A, FCER1A, CD1E, HLA*-*DQB2, S100B*). There was a non-significant tendency towards an increase in cDC1 (marked by *CADM1, SIPA1L3, CLEC9A, WDFY4, HDAC9*) or mature DC populations (marked by *CCL19, LAD1, CCR7, LAMP3, NCCRP1*) (Fig. 4F and fig. S14C). This is similar to our observation of DC accumulation in WT mouse lungs following bleomycin challenge, but not in *Mr1*^−/−^ mice, suggesting a potential role of DCs in modulating IPF-associated inflammation and fibrosis.

## Discussion

In this study of sterile lung injury, we uncover a novel role for MAIT cells; they are activated and enhance pulmonary accumulation of CD103^+^ cDC1, which limit pathology via DNGR-1. Consistent with these findings, scRNA-seq data from IPF patients reveal activated MAIT cells, and increased cDC populations in the lungs of IPF patients compared with controls. Our observations demonstrate the potential of MAIT cells as important orchestrators of tissue protection and modulators of inflammatory disease pathology.

MAIT cell activation in response to inflammation can occur through MR1-TCR-dependent pathways, typically for bacterial defense, or MR1-TCR-independent pathways, mediated by interleukins (IL-12/-15/-18) and type I interferon, linked to antiviral responses ^37^. In our study, lung MAIT cells showed earlier and more intense CD69 upregulation compared to non-MAIT αβ T cells, underscoring their rapid response to sterile injury. MAIT cell accumulation and CD69 upregulation were significantly reduced in both *Il18r1*^−/−^ and *Ifnar1*^−/−^ mice. This aligns with our previous findings from murine influenza studies ^13^, suggesting a strongly cytokine-driven response, dominated by IFN-α, in this model of sterile challenge.

In our research, we have observed a consistent downregulation of *Cd53* and *Gzma* in MAIT cells under various conditions. Specifically, in mice, MAIT cells exhibit reduced *Cd53* expression seven days post-*L. longbeachae* infection and decreased *Gzma* levels following acute infection compared to re-infected mice. Similarly, in humans, MAIT cells stimulated with 5-OP-RU demonstrate diminished *GZMA* expression relative to unstimulated cells ^6^. Cd53 is a key tetraspanin involved in cell adhesion, signalling, and immune interactions, essential for robust immune responses ^60^. It has been suggested that a deficiency in this protein heightens susceptibility to various pathogens, leading to recurrent viral, bacterial, and fungal infections in affected individuals ^61^. The observed decrease in *CD53* in MAIT cells suggests a functional shift that could increase vulnerability to cell death induced by bleomycin challenge. Conversely, the reduction in *Gzma*, a principal cytolytic enzyme ^62^, appears to be a regulatory adjustment aimed at minimising tissue damage during inflammatory responses, potentially steering MAIT cells towards a more regulatory role. This adaptive reprogramming may serve to balance immune defence mechanisms with the need for tissue preservation.

After bleomycin exposure, *Mr1*^−/−^ mice showed exacerbated weight loss and increased gene expression of *Col1a1* and *Col3a1*, suggesting intensified lung tissue. This aligns with findings across various models where *Mr1*^−/−^ mice exhibit compromised tissue integrity, suggesting MAIT cells play a protective role in maintaining barrier homeostasis ^4,7,63^. For instance, in type 1 diabetes and graft-versus-host disease models, *Mr1*^−/−^ mice demonstrated worsened disease outcomes due to impaired barrier functions ^64,65^. Additionally, in a model of non-alcoholic steatohepatitis, these mice suffered more severe liver damage, potentially due to imbalanced macrophage responses ^66^. Recent studies also show that MAIT cells in the meninges protect against oxidative stress and cognitive impairment by regulating antioxidant levels and preventing barrier leakage ^67^. These observations collectively underscore MAIT cells’ crucial role in preserving tissue integrity and mitigating inflammatory damage.

Although MAIT cells have never been assessed in pulmonary fibrosis, murine skin resident MAIT cells exhibit a distinct tissue repair transcriptional signature^4^, similar to H2-M3 restricted CD8^+^ T cells ^68^, and seen in TCR-activated MAIT cells in humans and mice ^6,7,69^, indicating their role in local repair similar to other tissue-resident cells. In models of collagen-induced arthritis^70^ and chronic liver injury^71^, MAIT cells intensified inflammation and pathology; their absence reduced these conditions. Pharmacological inhibition of MAIT cells also alleviated liver fibrosis, indicating interactions with monocytes/macrophages ^72^.

Significant weight loss differences were noted between *Mr1*^−/−^ and WT mice following bleomycin challenge, with a delayed decrease in cDC1 accumulation in *Mr1*^−/−^ mice evident by day 7. This suggests a broader role for MAIT cells in tissue homeostasis beyond the cDC1-DNGR1 pathway. Our scRNA-seq data reveal baseline differences between *Mr1*^−/−^ and WT mice, including fewer alveolar macrophages and lower *Pbx1* expression in *Mr1*^−/−^ mice (fig. S9 and S10, data S4), which is important in mitigating inflammation via IL10 transcription during apoptosis ^73–75^. This supports the notion of MAIT cells promoting an anti-inflammatory state in alveolar macrophages. Additionally, early upregulation of pro-inflammatory genes, *Il6* and *Ccl2* in *Mr1*^−/−^ mice corresponds with initial weight loss, with mast cells, interstitial macrophages, fibroblasts and CCR2^+^ Ly6C^hi^ monocytes identified as primary sources (fig. S15).

MAIT cells and DCs collaborate to orchestrate immune responses against pathogens and maintain immune protection. In mouse models, MAIT cells influenced DC maturation via GM-CSF during *Francisella tularensis* infection, though they were not directly identified as the source ^11^. Human studies showed that co-culturing MAIT cells with immature DCs in the presence of 5-Amino-6-D ribitylaminouracil/methylglyoxal (5-A-RU/MeG), led to MR1-dependent DC maturation marked by increased expression of CD86, CD80, CD40, and PD-L1, IL-12 production reliant on MR1 and CD40L^76^. *In vivo*, pulmonary MAIT cell stimulation with 5-A-RU/MeG or CpG triggered CD11b^+^ DC accumulation in the lung and their migration to the mediastinal lymph node. The exact mechanism behind DC accumulation post-MAIT cell activation remain unexplored ^12^. We have also shown MAIT cells enhance early immune response to adenovirus vector vaccines, requiring pDC-derived IFN-α, monocyte-derived IL-18, and TNF ^14^. Moreover, intranasal immunisation with MAIT cell agonists activates DCs via CD40L, primes T follicular helper cells and induces protective humoral immunity, suggesting their potential as adjuvants in mucosal vaccines ^77^.

In our investigation, we found that *Mr1*^−/−^ mice demonstrated an impaired early accumulation of CD103^+^ cDC1 and pre-DC populations in the lungs upon bleomycin challenge, highlighting a significant role for MAIT cells in early immune responses to lung injury. Despite minimal differences in gene expression between WT and *Mr1*^−/−^ mice, our results suggests that MAIT cells are essential for the recruitment of cDC1 cells, though they do not alter DC’s functional traits. The exact mechanisms are still unclear; it is unknown if MAIT cells directly recruit DCs from the bloodstream or if they affect precursor cell populations. We also discovered that MAIT cells produce cytokines like GM-CSF and IFN-γ (fig. S3D), which are important for the maturation and migration of DCs to inflammation sites ^78,79^. Our study further showed an increase in inflammatory chemokines such as *Ccrl2, Ccl3*, and *Cxcl2* in MAIT cells following injury, which could help explain the migration and accumulation of DCs. These findings suggest a complex interplay where MAIT cells indirectly influence lung tissue response to injury through cytokine and chemokine pathways, offering insights into potential therapeutic targets for enhancing immune response in lung diseases.

Our scRNA-seq dataset reveals proportional differences in the ILC3 population between *Mr1*^*−/−*^ and WT mice, showing a notably lower frequency of ILC3 at baseline and impaired accumulation of ILC3 in *Mr1*^*−/−*^ mice during sterile injury. This suggests that MAIT cells may play an important role in regulating ILC3 numbers. ILC3s are known to regulate the activity of various immune cells including DCs, macrophages, eosinophils, and neutrophils, contributing to their recruitment, movement, and tissue reparative functions. In steady-state conditions, ILC3s secrete Th17-associated cytokines such as IL-17A, IL-17F, IL-22, and GM-CSF, while during inflammatory responses, they are also capable of producing IFN-γ ^80,81^. These cytokines interact with respective receptors – IL-17R, IL-22R, GM-CSFR and IFN-γR – on myeloid and stromal cells, which are particularly receptive to GM-CSF ^82,83^. The GM-CSF produced by ILC3s has been implicated in bridging innate and adaptive immunity through its influence on myeloid cells ^84^. IL-22 is expressed at barrier surfaces and plays a vital role in the maintenance of normal barrier homeostasis ^85^. It has been demonstrated that ILC3s detect damage-induced cell death, which in turn triggers IL-22-dependent tissue repair ^86^. Thus, MAIT cells might influence ILC3s, which facilitate tissue repair directly through the production of various cytokines and chemokines, or indirectly by affecting the migration and functionality of DCs, thereby limiting tissue damage. We also noted a trend towards increased proportions of iNKT cells, ciliated cells, and fibroblasts in the lungs of WT mice, a trend not observed in *Mr1*^−/−^ mice. The presence of iNKT cells in WT mice might contribute to mitigating bleomycin-induced pulmonary fibrosis through mechanisms such as downregulating TGF-β ^87^ and inhibiting IL-4-driven M2 macrophage polarization ^88^. The absence of a similar increase in ciliated cells and fibroblasts in *Mr1*^−/−^ mice suggests potential impairments in mucociliary clearance, epithelial integrity, and tissue repair processes. This could lead to compromised wound healing and exacerbated fibrosis in *Mr1*^−/−^ mice.

In our study, we investigated the function of MAIT cells in bleomycin-induced lung injury using a *Salmonella* Typhimurium pre-infected, MAIT cell-boosted mouse model. This model was selected because it facilitates clearer distinctions between WT and *Mr1*^*−/−*^ mice, without requiring a sublethal dose or resulting in mortality and thus higher reproducibility, and better mirrors the MAIT cell frequencies and phenotypes observed in human lungs. This approach was originally described by Z. Chen *et al* ^30^ and was recently utilised by T. Riffelmacher *et al*. ^31^, aligning with established methodologies for studying MAIT cell function *in vivo*. MAIT cells boosted by *Salmonella* Typhimurium BRD509 exhibit a hybrid MAIT1/MAIT17 phenotype ^30,31,33^, which exists in *Homo sapiens* as well as *Monodelphis domestica* (opossum), *Bos taurus* (cattle), *Ovis aries* (sheep), but not in *Rattus norvegicus* (rat) *and Mus musculus*, suggesting that *Salmonella* Typhimurium BRD509-boosted MAIT cells in mouse captured the phenotype in human better ^36^. To ensure that our findings were not confounded by baseline differences between the genotypes, both WT and *Mr1*^*−/−*^ mice underwent identical *Salmonella* Typhimurium treatments prior to bleomycin challenge. Rigorous validation using spectral flow cytometry, bulk RNA-seq, and scRNA-seq showed minimal transcriptomic and phenotypic differences between the two genotypes before bleomycin exposure. Therefore, the observed post-challenge differences were attributed to the presence or absence of MAIT cells expanded by *Salmonella* Typhimurium infection, not confounded by the pre-infection.

Our study indicates that MAIT cells are notably activated in the lungs of IPF patients compared to non-fibrotic controls, with limited literature on their role in IPF thus far. Previous research indicates a decrease in MAIT cells counts in IPF patients’ blood, suggesting a potential migration to the lungs ^89^. Future research could investigate MAIT cells’ roles in pulmonary fibrosis and their interactions with lung microbiota, given their response to microbial infections.

Using the bleomycin-challenged murine model, a well-accepted surrogate of interstitial lung disease (ILD) ^90^, we have identified an important role for MAIT cells in mitigating lung injury, providing key insights into the mechanisms of pulmonary homeostasis. Common pathological traits like airway damage and tissue remodelling, which significantly contribute to the progression of various respiratory disorders ^91^ , underscore the potential of our findings in developing novel antifibrotic therapies. Strategies could include enhancing MAIT cell activity via synthetic, riboflavin-competent commensal organisms ^92^, or targeting the DNGR-1 pathway. Further studies are needed to explore MAIT cells’ roles in human lung injury, and to validate the translational potential of our *in vivo* findings.

### Limitations of the study

We performed adoptive transfer of FLT3L-BMDCs into *Mr1*^*−/−*^ mice and demonstrated that MAIT cells indirectly promote the accumulation of DCs to mediate tissue repair. Ideally, we would have confirmed MAIT cells’ direct role through adoptive transfer into *Mr1*^−/−^ mice; however, technical and ethical constraints within our facility made this impractical. Challenges included limitations with our specific strain of *Salmonella* Typhimurium BRD509, difficulties in isolating sufficient numbers of MAIT cells, and ethical constraints, particularly restricting weight loss to less than 20% for a limited duration. However, it is important to note that we have not claimed a direct effect of MAIT cells on tissue repair; rather, we suggest that their role is mediated by influencing DC accumulation, which in turn affects tissue repair.

Moreover, while we noted a transient increase in DCs, particularly cDC1, in WT mice after a bleomycin challenge, data from human IPF patients show a more pronounced accumulation of cDC2, indicating species differences in immune responses. These discrepancies highlight the challenges of extrapolating mouse model findings to human disease, as mouse models do not fully replicate the chronic progression of human IPF. The variation in DC dynamics between species underscores the complexities in translating fibrosis studies from mice to humans and suggests that immune responses, including DC activation, may evolve differently across the progression of fibrosis.

## Resource availability

### Lead Contact

Requests for further information and resources should be directed to and will be fulfilled by the lead contact, Timothy Hinks, Respiratory Medicine Unit, Experimental Medicine Division, Nuffield Department of Medicine, University of Oxford, John Radcliffe Hospital, Oxford, OX3 9DU, United Kingdom (timothy.hinks@ndm.ox.ac.uk).

### Materials Availability

This study did not generate new unique reagent. All data needed to evaluate the conclusions in the paper are present in the paper and/or the Supplementary Materials.

## STAR★Methods

### Key Resources Table

**Table:** 

REAGENT or RESOURCE	SOURCE	IDENTIFIER
**Antibodies**		
AF700 anti-mouse CD103	BioLegend	Cat# 121442; RRID: AB_2813992
BUV395 anti-mouse CD45	BD Biosciences	Cat# 564279; RRID: AB_2651134
APC/Fire™ 750 anti-mouse CD11c	Biolegend	Cat# 117352; RRID: AB_2572124
BV480 anti-mouse CD19	BD Biosciences	Cat# 566107; RRID: AB_2739509
PE-Cy7 anti-mouse MERTK (Mer)	Biolegend	Cat# 151522; RRID: AB_2876508
AF488 anti-mouse CD4	Biolegend	Cat# 100423; RRID: AB_389302
BUV615 anti-mouse Ly-6G	BD Biosciences	Cat# 751263; RRID: AB_2875279
BV570 anti-mouse Ly-6C	Biolegend	Cat# 128029; RRID: AB_10896061
BV650 anti-mouse MHC II	Biolegend	Cat# 107641; RRID: AB_2565975
BV605 anti-mouse CD8a	Biolegend	Cat# 100744; RRID: AB_2562609
BV711 anti-mouse CD11b	Biolegend	Cat# 101242; RRID: AB_2563310
BV785 anti-mouse CD3	Biolegend	Cat# 100232; RRID: AB_11218805
PE anti-mouse CD64	Biolegend	Cat# 139304; RRID: AB_10613467
PE/Cyanine5 anti-mouse NK1.1	Biolegend	Cat# 108716; RRID: AB_493590
BUV805 anti-mouse CD44	BD Biosciences	Cat# 741921; RRID: AB_2871234
BUV737 anti-mouse TCR γ/δ	BD Biosciences	Cat# 748991; RRID: AB_2873389
PerCP Cy5.5 anti-mouse CD19	BioLegend	Cat# 115534; RRID: AB_2072925
FITC anti-mouse CD69	BD Biosciences	Cat# 553236; RRID: AB_396675
PE-Cy7 anti-mouse TCRβ	BD Biosciences	Cat# 560729; RRID: AB_1937310
APC anti-mouse CD25	eBioscience™	Cat# 17-0251-82; RRID: AB_469366
BV711 anti-mouse CD45.2	BD Biosciences	Cat# 563685; RRID: AB_2738374
BV650 anti-mouse IFN-γ	BD Biosciences	Cat# 563854; RRID: AB_2738451
APC anti-mouse IL-22	BioLegend	Cat# 516409; RRID: AB_2563355
APC-Cy7 anti-mouse IL-10	BioLegend	Cat# 505010; RRID: AB_315363
PE anti-mouse IL-17A	BioLegend	Cat# 506904; RRID: AB_315463
PE/Dazzle™ 594 anti-mouse GM-CSF	BioLegend	Cat# 505422; RRID: AB_2814425
PerCPCy5.5 anti-mouse SiglecH	BioLegend	Cat# 129614; RRID: AB_10639936
FITC anti-mouse CD45	BioLegend	Cat# 103108; RRID: AB_312972
BV605 anti-mouse CD86	BioLegend	Cat# 105037; RRID: AB_11204429
PE/Cyanine5 anti-mouse CD40	BioLegend	Cat# 124618; RRID: AB_2075922
PE/Dazzle™ 594 anti-mouse CD192	BioLegend	Cat# 150636; RRID: AB_2922471
APC/Fire™ 750 anti-mouse CD192	BioLegend	Cat# 150630; RRID: AB_2810416
PE/Cyanine7 anti-mouse CD317	eBioscience™	Cat# 25-3172-82; RRID: AB_2573440
APC anti-mouse CD370	BioLegend	Cat# 143506; RRID: AB_2566379
PE/Dazzle™ 594 anti-mouse CD24	BioLegend	Cat# 101838; RRID: AB_2566732
FITC anti-mouse CD45.2	BioLegend	Cat# 109806; RRID: AB_313442
BV785 anti-mouse CD45.1	BioLegend	Cat# 110743; RRID: AB_2563379
PE anti-mouse CD11c	BioLegend	Cat# 117308; RRID: AB_313776
BV421 anti-mouse CD45R	BioLegend	Cat# 103251; RRID: AB_2562905
*InVivo*MAb anti-mouse CLEC9A (CD370)	BioXCell	Cat# BE0305
*InVivo*MAb rat IgG1 isotype control, anti-horseradish peroxidase	BioXCell	Cat# BE0088
*InVivo*Pure pH 7.0 Dilution Buffer	BioXCell	Cat# IP0070
**Bacterial and virus strains**		
*Salmonella enterica* serovar Typhimurium, strain BRD509	Gordon Dougan, Sanger Centre, Cambridge^93^	N/A
**Chemicals, peptides, and recombinant proteins**		
Murine MR1-5-OP-RU monomers	NIH Tetramer Facility	N/A
Murine MR1-6-FP monomers	NIH Tetramer Facility	N/A
Brilliant Violet 421 (BV421)-Streptavidin	BioLegend	Cat# 405225
Phycoerythrin (PE)-Streptavidin	BioLegend	Cat# 405245
Brefeldin A	eBioscience™	Cat# 00-4506-51
IC fixation buffer	eBioscience™	Cat# 00-8222-49
Permeabilization buffer	eBioscience™	Cat# 00-8333-56
Percoll	Cytiva	Cat# 17-0891-01
Zombie NIR™ Fixable Viability Kit	BioLegend	Cat# 423106
Zombie Aqua™ Fixable Viability Kit	BioLegend	Cat# 423102
Zombie Yellow™ Fixable Viability Kit	BioLegend	Cat# 423104
RNeasy Plus Micro Kit	QiaGen	Cat# 74034
High Sensitivity RNA ScreenTape	Agilent	Cat# 5067-5579
High Sensitivity RNA ScreenTape Sample Buffer	Agilent	Cat# 5067-5580
High Sensitivity RNA ScreenTape Ladder	Agilent	Cat# 5067-5581
NEBNext® Ultra™ II Directional RNA Library Prep Kit	NEB	Cat# 7530
TaKaRa SMART-Seq v4 Ultra Low Input RNA Kit	TaKaRa	Cat# 634889
anti-PE microbeads	Miltenyi Biotec	Cat# 130-048-801
Formalin solution, neutral buffered, histological tissue fixative	Sigma-Aldrich	Cat# HT501128-4L
Ethyl alcohol, pure	Sigma-Aldrich	Cat# 1085430250
Histo-Clear II	Scientific Laboratory Supplies	Cat# NAT1334
Paraffin wax	Sigma-Aldrich	Cat# 76242
Trichrome Stain Kit	Abcam	Cat# ab150686
Hydroxyproline Assay Kit	Sigma-Aldrich	Cat# MAK008
High-Capacity cDNA Reverse Transcription Kit with RNase Inhibitor	Applied Biosystems	Cat# 4374966
QuantiFast® SYBR® Green PCR Kit	QiaGen	Cat# 204056
**Deposited data**		
scRNA-Seq of lungs from *Salmonella* Typhimurium BRD509-boosted WT and *Mrl*^−/−^ mice challenged with bleomycin	Gene Expression Omnibus	GSE270870
Bulk RNA-seq of lung MAIT cells or total lungs from *Salmonella* Typhimurium BRD509-boosted WT and *Mrl*^−/−^ mice challenged with bleomycin	Gene Expression Omnibus	GSE270725
Bulk RNA-seq of lung MAIT cells from WT and *Mrl*^−/−^ mice challenged with bleomycin	Gene Expression Omnibus	GSE270623
IPF dataset from Habermann et al. ^55^	Gene Expression Omnibus	GSE135893
IPF dataset from Adams et al. ^56^	Gene Expression Omnibus	GSE136831
**Experimental models: Organisms/strains**		
Mouse C57BL/6	University of Oxford Biomedical Services (BMS), Charles River or Envigo	MGI ID: 3028467
Mouse *Mrl*^−/−^	University of Oxford	MGI ID: 3664578
Mouse *Il18ri^tm1Aki^*	University of Oxford	MGI ID: 2136765
Mouse *Ifnar1^tm1Agt^*	University of Oxford	MGI ID: 1930950
Mouse B6.SJL-Ptprc^a^ Pepc^b^/BoyJ	University of Oxford Biomedical Services (BMS)	MGI ID: 2164701
**Oligonucleotides**		
*Actb* forward primer (5’ > 3’) TCC ATC ATG AAG TGT GAC GT	Life Technologies	Self-designed
*Actb* reverse primer (5’ > 3’) GAG CAA TGA TCT TGA TCT TCA T	Life Technologies	Self-designed
*Collai* forward primer (5’ > 3’) GCTCCTCTTAGGGGCCACT	Life Technologies	Self-designed
*Collai* reverse primer (5’ > 3’) CCACGTCTCACCATTGGGG	Life Technologies	Self-designed
*Col3ai* forward primer (5’ > 3’) CTGTAACATGGAAACTGGGGAAA	Life Technologies	Self-designed
*Col3ai* reverse primer (5’ > 3’) CCATAGCTGAACTGAAAACCACC	Life Technologies	Self-designed
**Software and algorithms**		
SpectroFlo® version 3.0	Cytek Biosciences	https://cytekbio.com/blogs/resources/spectroflo-v3-0-software-release-notes
FlowJo version 10.8.1	FlowJo, LLC	https://docs.flowjo.com/flowjo/getting-acquainted/10-8-release-notes/10-8-1-release-notes/
Prism version 9.2.0	GraphPad	https://www.graphpad.com/updates/prism-920-release-notes
RStudio version 1.4.1717	R Consortium	https://posit.co/products/open-source/rstudio/
STAR 2.6.1	Dobin et al. ^94^	https://github.com/alexdobin/STAR
featureCounts 1.5.0	Liao et al. ^95^	https://subread.sourceforge.net/featureCounts.html
DESeq2 1.30.1	Love et al. ^96^	https://bioconductor.org/packages/release/bioc/html/DESeq2.html
clusterProfiler 4	Wu et al. ^97^	https://guangchuangyu.github.io/software/clusterProfiler/
Mfuzz	Kumar et al. ^98^	http://mfuzz.sysbiolab.eu/
GSEA 4.1.0	Subramanian et al. ^99^	https://www.gsea-msigdb.org/gsea/index.jsp
CellRanger suite 7.1.0	10x Genomics	https://www.10xgenomics.com/support/software/cell-ranger/latest
Seurat 4.1.0	Hao et al. ^100^	https://satijalab.org/seurat/
CITEviz 0.99.0	Kong et al. ^101^	https://github.com/maxsonBraunLab/CITEViz
dsb 1.0.2	Mule et al. ^102^	https://github.com/niaid/dsb
celda 1.10.0	Yang et al. ^103^	https://github.com/campbio/celda
Scanpy 1.9.1	Wolf et al. ^104^	https://scanpy.readthedocs.io/en/stable/
Scrublet 0.2.3	Wolock et al. ^105^	https://github.com/swolock/scrublet
harmonypy 0.0.6	Korsunsky et al. ^106^	https://github.com/slowkow/harmonypy
umap-learn 0.5.3	Becht et al. ^107^	https://umap-learn.readthedocs.io/en/latest/
leidenalg 0.8.10	Traag et al. ^108^	https://github.com/vtraag/leidenalg
edgeR 3.36.0	Robinson et al. ^109^	https://bioconductor.org/packages/release/bioc/html/edgeR.html
limma 3.50.3	Ritchie et al. ^110^	https://bioconductor.org/packages/release/bioc/html/limma.html
Monocle 2.22.0	Qiu et al. ^50^	https://cole-trapnell-lab.github.io/monocle-release/docs/

## Method Details

### Mice model and *in vivo* bleomycin challenge

C57BL/6 mice (aged 8–10 weeks) were purchased from University of Oxford Biomedical Services (BMS), Charles River or Envigo. *Mr1*^−/−^ mice ^111^ (kindly provided by Dr Claire Hutchings, University of Oxford, MGI ID: 3664578), *Il18r1*^*tm1Aki*^ mice (kindly provided by Prof Kevin Maloy, University of Oxford, MGI: 2136765), and *Ifnar1*^*tm1Agt*^ mice (kindly provided by Dr Claire Hutchings, University of Oxford, MGI ID: 1930950) were bred in house and used at 8-10 weeks of age. Both male and female mice were used across all experimental groups, including C57BL/6, *Mr1*^−/−^, *Il18r1*^*tm1Aki*^, and *Ifnar1*^*tm1Agt*^ strains. Sex and age were matched within each comparison group to ensure the minimisation of potential biases and enhance the reliability of our findings. All mice were housed in specific pathogen-free conditions. For indicated experiments, C57BL/6 and *Mr1*^−/−^ mice were co-housed for ≥ 28 days to normalize the microbiome between strains ^65^. All work was performed under UK Home Office license PPL P61FAD253 or PP1874135 in accordance with the UK Animal (Scientific Procedures) Act 1986. All work was performed by trained and licensed individuals. For the bleomycin challenge, mice were anaesthetized with isoflurane and treated intratracheally with 1.875 U/Kg (mice weight) of bleomycin sulphate (Apollo Scientific, Cat. No. BI3543) in 50 μL of PBS for *Salmonella* Typhimurium BRD509 pre-infected mice, or with 1.0 U/Kg of bleomycin sulphate in 50 μL of PBS for naïve mice without pre-infection.

### Mice pulmonary MAIT cell expansion using *Salmonella* Typhimurium BRD509

*Salmonella* Typhimurium BRD509 were prepared as previously described ^13^. Mice were infected intranasally with 10^6^ CFU *Salmonella*. Typhimurium BRD509 in 50 μL PBS under isoflurane anaesthesia.

### Generation of MR1 tetramers

Murine MR1-5-OP-RU and MR1-6-FP monomers were provided by the NIH Tetramer Facility. Tetramers were generated using Brilliant Violet 421 (BV421)-Streptavidin and Phycoerythrin (PE)-Streptavidin (BioLegend, Cat. No. 405225 and 405245, respectively) following the NIH Tetramer Facility’s guidelines.

### Antibodies staining for flow cytometry and cell sorting

Murine lung tissues were prepared as described previously ^6^. For measurement of intracellular markers, 1 × Brefeldin A (eBioscience™, Cat. No. 00-4506-51) was added 4 hours before staining. Lung cells were blocked with anti-Fc receptor 2.4G2 and/or 6-FP tetramer for 15 min at room temperature (RT), stained with viability dye, fluorescently labelled MR1 tetramer and/or flow cytometric antibodies for 20 min at RT. Staining antibodies, clones and concentrations are listed in table S2. Samples were washed in FACS buffer (PBS + 0.5% BSA + 2 mM EDTA), and cells were fixed for 15 min RT using IC fixation buffer then washed twice with 1 × permeabilization buffer (eBioscience™, Cat. No. 00-8222-49 and 00-8333-56, respectively). Intracellular staining was performed overnight at 4°C. Samples were subsequently washed twice and stored in FACS buffer at 4°C until analysed on BD LSRII flow cytometer.

For live cell sorting on murine lung MAIT cells, lung single-cell suspension was purified with a 40%: 70% Percoll gradient. The sorting was conducted on a BD Aria III directly into a 350µL lysis buffer (Buffer RLT plus, supplemented with 10µL β-ME per 1mL Buffer RLT plus from the QiaGen RNeasy Plus Micro Kit, Cat. No. 74034) and subsequently stored at -80 °C for future batch RNA extraction.

For multiparameter spectral flow cytometry analysis, we used the “AF as a tag” (AF) function in the SpectroFlo (Cytek Biosciences, CA) software ^112^. 6 unique AF tags were disassociated from unstained mouse lung samples and included in the unmixing strategy.

### Total RNA extraction and RNA integrity assessment

RNA extraction was performed by single column centrifugation using the RNeasy® Plus Micro Kit (Qiagen, Cat No. 74034) following the manufacturer’s protocol. RNA integrity was assessed by Agilent High Sensitivity RNA ScreenTape Assay on an Agilent 4200 TapeStation following the manufacturer’s protocol (Agilent, Cat. No. 5067-5579, 5580 and 5581).

### mRNA isolation, library preparation, sequencing by Novogene

Total RNA was subsequently submitted to Novogene following their sample preparation and shipping instructions. All further laboratory work was performed by Novogene using their commercial protocol. RNA was reverse transcribed, and cDNA amplified with the NEBNext® Ultra™ II Directional RNA Library Prep Kit for Illumina® (NEB, Cat. No. E7760S), a low input method using NEB Next® Ultra RNA Library Prep Kit for Illumina® (NEB, Cat. No. 7530), or an ultra-low input method using TaKaRa SMART-Seq v4 Ultra Low Input RNA Kit for Sequencing (TaKaRa, Cat. No. 634889). First-strand cDNA synthesis and tailing by reverse transcription were performed using SMART (Switching Mechanism at 5’ End of RNA Template) technology. Following first-strand synthesis, cDNA was amplified by PCR to produce the library. Quality control of the library was performed by quantification with a Qubit 2.0 fluorimeter and by qPCR. Insert size was measured by the Agilent 2100 Bioanalyzer automated gel electrophoresis system. The library was sequenced using the NovaSeq platform with Illumina sequencing technology to generate 150bp paired-end reads.

### RNA-sequencing data analysis

NovaSeq platform images were first converted into raw sequence reads via Illumina’s CASAVA software, stored in FASTQ format. After filtering out low-quality and adapter reads, the remaining clean reads were mapped using STAR version 2.6.1 ^94^ against the mus musculus GRCm38 reference genome (GenBank accession number GCA_000001635.2). Successful mapping was determined by a rate over 70%, with the results preserved as BAM files ^113^. Read quantification involved featureCounts 1.5.0 ^95^, which converted BAM files to a table of gene IDs and counts per sample. Differential expression analysis was performed in in R (version 4.1.0) using DESeq2 (version 1.30.1) ^96^. DEGs were defined as log2 fold-change > 1 and adjusted *P* < 0.05. VennDiagram (version 1.6.20). And ggplot2 (version 3.2.1), pheatmap (version 1.4.3) and ggrepel (version 0.8.1) were used for data visualization. clusterProfiler (version 4.0) ^97^ and Mfuzz ^98^ are used for GO enrichment and time-series analysis, respectively. GSEA was performed using GSEA software (version 4.1.0) ^99^.

### 10x Genomics library generation, sequencing and computational analysis

Sequencing libraries were generated using 10x Genomics Chromium Next GEM Single Cell 5’ Reagent kit v2 (Dual Index) following manufacturer’s instructions (CG000330 Rev D). ADT-labelled (BioLegend, Cat. No. 155861, 155863 and 199903) cells were loaded onto the Chromium iX (10x Genomics) at a concentration of ~1 x 10^6^ cells/mL, with 50,000 cells loaded per channel. One channer was loaded per 2 mice lung samples. Library generation was performed using Biomek FX^P^ Laboratory Automation Workstation (Beckman Coulter) at MRC Weatherall Institute of Molecular Medicine Single-Cell Facility (WIMM, University of Oxford). Library quality and concentration was assessed using a Bioanalyzer (Agilent) and Qubit 2.0 Fluorometer (Thermo Fisher Scientific), respectively. Libraries were sequenced on an Illumina NovaSeq 6000 to a mean depth of 40,000 read pairs/cell for scRNA-seq, 10,000 read pairs/cell for Cite-seq and TCR-seq, performed at Novogene.

10x Genomics cellranger analysis pipelines were used to generate single cell gene counts. Reads from gene expression and TCR library were aligned to the mouse mm10 reference genome (version 2020-A) and GRCm38 Mouse V(D)J Reference-7.0.0 (May 17, 2022), respectively, and quantified using cellranger multi pipeline together with those from ADT library.

Hashtag oligo (HTO) data underwent a transformation using Seurat’s centred log-ratio (CLR) transformation ^100^. Demultiplexing of HTO hashtags was subsequently performed manually with CITEviz ^101^, followed by normalisation of CITE-seq data via the dsb package ^102^. Ambient RNA was removed using decontX ^103^. The RNA-seq data were then processed using Scanpy ^104^, which involved doublet removal with Scrublet ^105^ and cell filtering with specified thresholds for total counts (1,000 – 60,000), genes by counts (500 – 6,000), and mitochondrial counts (0 – 10%). The filtered data underwent normalization to achieve a total sum of 10,000 and were log-transformed with a pseudocount of 1. Highly variable genes were identified by setting the flavour to “cell_ranger”. Principal Component Analysis (PCA) was conducted, with the number of principal components set using the KneeLocator function. Cells from different mice were subsequently integrated using the Harmony algorithm ^106^. Neighbourhoods were identified with n_neighbors set to 5, followed by dimensionality reduction with UMAP ^107^ and partitioning cell type with Leiden clustering at a resolution of 2.0 ^108^. DEGs between cell types were identified using the rank_genes_groups function with a t-test. Cell clusters were identified using both RNA and protein expression data. T cells were further selected for reintegration and subset identification using the same method as previously stated, except that the clustering resolution was set to 1.0.

PCA on the overall transcriptome for each mouse was based on pseudobulk counts, computed by summing the counts of all cells in each mouse. Genes with low expression was filtered using filterByExpr in edgeR (version 3.36.0) ^109^ by setting min.count to 3, and using model matrix adjusted for time point and genotype. Normalisation factors were calculated using calcNormFactors in edgeR and the pseudocounts were then normalised using voom in limma (version 3.50.3) ^110^.

MAIT cells and iNKT cells were identified using clonotypes.csv and filtered_contig_annotations.csv, based on the output generated by cellranger multi pipeline. A cell is designated as a MAIT cell if it is part of a clonotype that exhibits *Trav1* and *Traj33* expression. A cell is designated as an iNKT cell if it is part of a clonotype that exhibits *Trav11* and *Traj18* expression.

Differential gene expression between *Mr1*^−/−^ and WT mice across various time points and cell types was analysed using DESeq2 ^96^ and pseudobulk counts, computed by summing the counts of all cells within each cell type for each mouse. Enriched gene sets were identified using the pre-ranked gene-set enrichment analysis (GSEA) algorithm implemented in the FGSEA R package ^114^. Genes were ranked with the log2 fold change for the relevant coefficient calculated by DESeq2. Enrichment was assessed with gene set list from MSigDB’s Hallmark collection.

In the trajectory analysis of DC populations, Monocle 2 ^50^ (version 2.22.0) was utilized. The raw count data was processed to establish a CellDataSet object within Monocle 2 by setting the expressionFamily to a negative binomial distribution with a fixed variance. Cell ordering was achieved using genes identified by dpFeature. Dimension reduction for visualization was carried out using DDRTree. Pre-cDCs were identified as the root_state during the cell ordering process. Trajectories were generated independently for cells from different groups. The expression profiles of selected genes in the two differentiation branches (cDC1 and cDC2) were visualized using the “plot_genes_branched_heatmap” function within the Monocle2 package ^50^.

### Analysis of human IPF scRNA-seq datasets in HLCA and IPF cell atlas

Cells within the HLCA and IPF cell atlas were assessed for *TRAV1-2* expression. Those exhibiting positive *TRAV1-2* expression levels were categorized as MAIT cells. The primary identification of MAIT cells was in GSE135893 ^55^, and only these MAIT cells were utilized in subsequent analyses. Differential gene expression between MAIT cells from IPF patients and those from healthy controls was determined using the FindMarkers function in Seurat ^100^, with a pseudocount set to 1. For the overrepresentation test, modules in BTM ^59^ were employed, utilizing the enricher function in clusterProfiler ^97^. Cell-cell interactions in both IPF patients and healthy controls were identified using the li.mt.rank_aggregate function in the LIANA package.

Both GSE135893 and GSE136831 ^55,56^ were analysed in terms of immune cell composition changes, given that both datasets contained more than three samples from the IPF and healthy control groups, respectively. Marker genes for DC populations were derived from the original publication.

### Culture and adoptive transfer of Flt3 ligand-generated bone marrow-derived dendritic cells

B6.SJL-Ptprc^a^ Pepc^b^/BoyJ mice (purchased from University of Oxford Biomedical Services (BMS), MGI ID: 2164701) were used as donor mice and all donor mice were infected with 10^6^ CFU *Salmonella* Typhimurium BRD509 4 weeks before harvesting bone marrow cells. FLT3L-BMDC were generated by culture in RPMI complete containing murine Flt3L at 200ng/mL and murine GM-CSF at 20ng/mL as previously described ^115^. DNGR-1 was highly expressed in the MHCII^+^ CD11c^+^ CD24^hi^ subsets of Flt3L BMDCs (fig. S13B), which correspond to the CD103^+^ subset of lung DCs. CD11c^+^ FLT3L-BMDCs were enriched using anti-PE microbeads (Miltenyi Biotec, Cat. No. 130-048-801). 5 × 10^5^ FLT3L-BMDCs were given into each recipient *Mr1*^−/−^ mouse intranasally ^116^ at day 1 post-bleomycin challenge. When necessary, mice were treated i.p. with 100 μg of 7H11 anti-DNGR-1 blocking antibody or isotype-matched control (BioXCell, Cat. No. BE0305 and BE0088, respectively). Injections were administered daily from day -1 to day 10 post-bleomycin challenge.

### Histology

The left lobes of the mice lungs were preserved in 10% neutral buffered formalin, sequentially dehydrated with an ethanol gradient, cleared with Histo-Clear II, and infiltrated with paraffin wax. Subsequently, paraffin-embedded sections (4 μm thick) of these lobes were stained with Masson’s trichrome (Abcam, Cat. No. ab150686) following manufacturer’s instructions. To evaluate the extent of fibrosis, the modified Ashcroft scoring system was employed for a semiquantitative analysis ^117^.

### Hydroxyproline assay

Hydroxyproline was measured using 10 mg of lung tissue using a hydroxyproline assay kit (Sigma-Aldrich, Cat. No. MAK008) per the manufacturer’s instructions.

### RNA quantification, purity check and reverse transcription

For RT-qPCR experiments, total lung RNA was extracted as described above. RNA quantity and quality were assessed using a Nanodrop 2000 (Thermo Scientific) following the manufacturer’s protocol. Isolated RNA was converted to cDNA in preparation for qPCR using a High-Capacity cDNA Reverse Transcription Kit with RNase Inhibitor (Applied Biosystems, Cat. No. 4374966) following manufacturer’s protocol. Template RNA and reagents were thawed on ice. The reverse transcription reaction mix was prepared and incubated in the Programmable Thermal Controller as the following steps: Step 1: 25°C, 10 minutes; Step 2: 37°C, 120 minutes; Step 3: 85°C, 5 minutes; Step 4: 4°C, ∞.

### Reverse transcription quantitative polymerase chain reaction (RT-qPCR)

For qPCR reactions, 2 × QuantiFast SYBR Green PCR Master Mix kit (QiaGen, Cat No. 204056) was used following the manufacturer’s instructions. PCR reaction mix was prepared, mixed, and appropriate volumes were dispensed into the wells of a PCR plate. Template cDNA was added to the individual wells containing the reaction mix. qPCR plate was in loaded into a Bio-Rad CFX96. qPCR was performed following the manufacturer (Bio-Rad)’s instructions. Thermal cycling conditions were set up as the following steps: PCR initial heat activation: 95°C, 5 minutes; 2-step cycling: Denaturation: 95°C, 10s; Combined annealing/extension: 60°C, 30s; 35-40 cycles in total. For all tests, *P* < 0.05 was considered statistically significant.

### Data analysis and statistics

Flow cytometry data were acquired on a Cytek Aurora (Cytek Biosciences) or BD LSRII Flow Cytometer (BD Biosciences) and processed in SpectroFlo® version 3.0 (Cytek Biosciences) or FlowJo version 10.8.1 (FlowJo, LLC). Data were analysed in Prism version 9.2.0 (GraphPad) and RStudio version 1.4.1717. For *in vivo* mouse data analysis, various tests were deployed as required, including unpaired *t* tests, Mann-Whitney tests, one-way ANOVA with Dunnett’s or Sidak’s multiple comparisons, Kruskal-Wallis with Dunn’s multiple comparisons, and two-way ANOVA with Sidak’s multiple comparisons, Holm-Sidak’s multiple comparisons or Fisher’s LSD test. A *P* value less than 0.05 was considered significant.

## Supplementary Material

Supplemental Table 1

Supplemental Table 2

Supplemental Table 3

Supplemental Table 4

Supplementary information

Supplementary Materials

## Data Availability

The bulk RNA-Seq dataset of MAIT cells from the lungs of mice challenged with bleomycin, without prior *Salmonella* Typhimurium BRD509 exposure, is available in the GEO database under accession code GSE270623. The bulk RNA-Seq datasets from the lungs of mice challenged with bleomycin with the prior *Salmonella* Typhimurium BRD509 exposure, are deposited under accession code GSE270725. The scRNA-seq dataset is accessible under accession code GSE270870. This paper does not report original code. Any additional information required to reanalyse the data reported in this work paper is available from the lead contact upon request.
