# Supplementary material for: MAIT cells protect against sterile lung injury
Source: Cell Rep. Author manuscript; Available in PMC 2025 Jul 17. (PMC7617896; doi:10.1016/j.celrep.2025.115275)
Supplement: Supplementary information [file EMS206836-supplement-Supplementary_information.pdf]

Supplemental Information

This SI includes:

Figs. S1 to S15

Tables S1 and S2

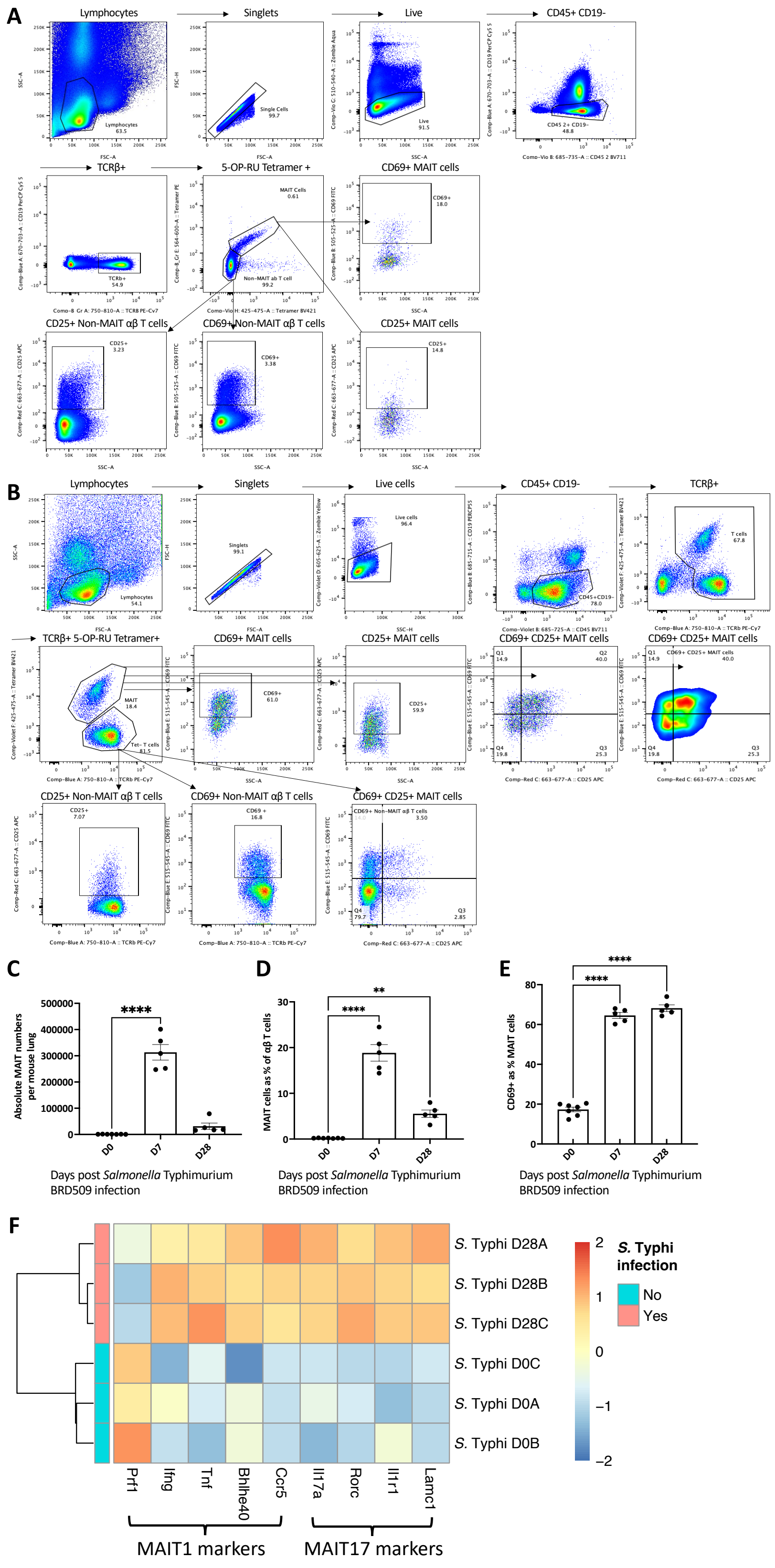

Fig. S1. Temporal dynamics of MAIT cells in pulmonary immune response to *Salmonella* Typhimurium infection; Related to Fig. 1. (**A** and **B**) Gating scheme for the identification of

MAIT cells (TCRβ$^+$ CD45.2$^+$ CD19$^-$ MR1-5-OP-RU tetramer$^+$ cells), and expression of CD25 and CD69 on MAIT and non-MAIT αβ T cells in naïve mice lung (**A**) and *Salmonella* Typhimurium BRD509 infected mice lung (**B**). (**C** and **D**) Bar plots showing the absolute MAIT cell numbers (**C**) and percentage of MAIT cells of total pulmonary αβ T cells (**D**) post BRD509 infection. (**E**) Proportion of pulmonary MAIT cells expressing CD69 expressed as a percentage of pulmonary MAIT cells post BRD509 infection. Data (mean ± S.E.M.) are one representative experiment of two independent experiments, with 5–7 mice per group in each replicate. Statistical significance tested by one-way ANOVA with Dunnett's multiple comparison tests. ** $P < 0.01$; **** $P < 0.0001$. (**F**) Heatmap showing relative expression of MAIT1 and MAIT17 marker genes in lung MAIT cells at Day 28 post- *Salmonella* Typhimurium infection, compared to Day 0.

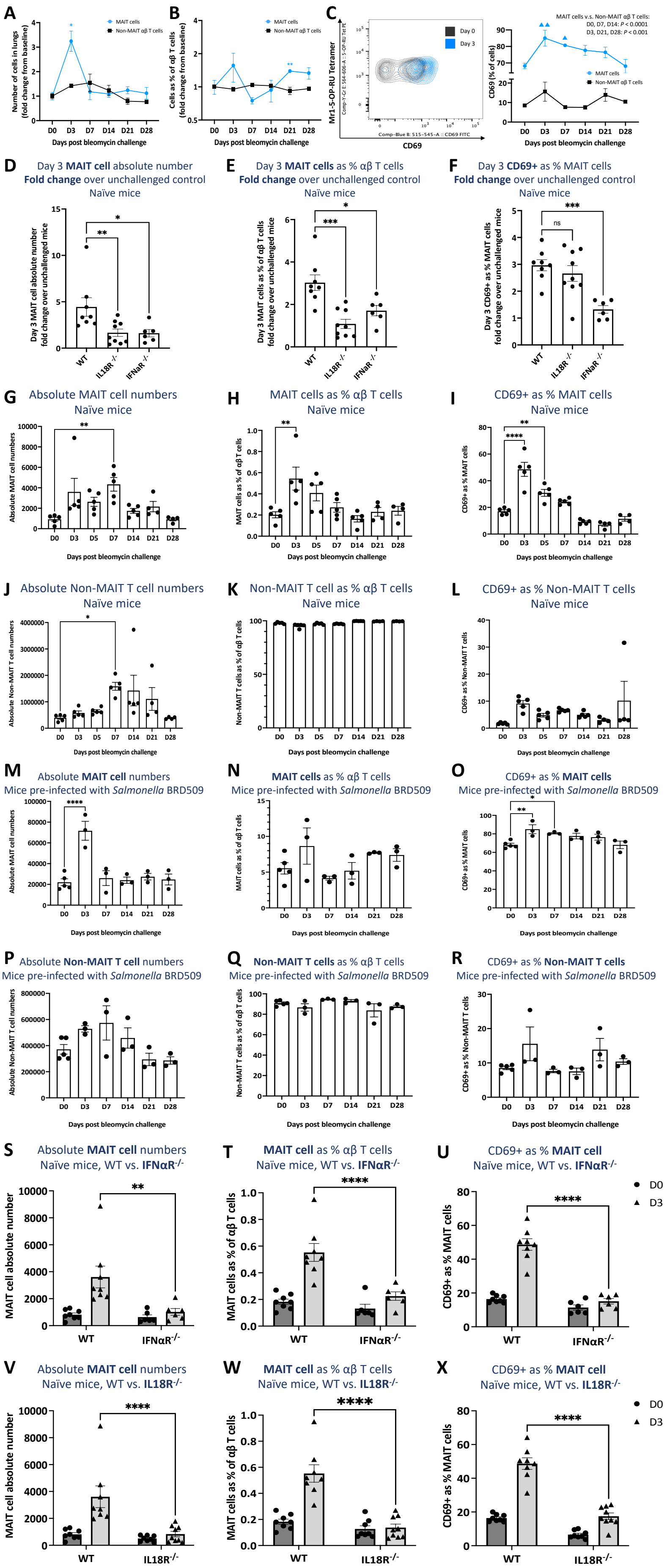
A
Number of cells in lungs (fold change from baseline)
MAIT cells
Non-MAIT αβ T cells
Days post bleomycin challenge
B
Cells as % of αβ T cells (fold change from baseline)
C
Mr1-5-OP-RU Tetramer
Day 0
Day 3
CD69
MAIT cells v.s. Non-MAIT αβ T cells:
D0, D7, D14: P < 0.0001
D3, D21, D28: P < 0.001
CD69 (% of cells)
D
Day 3 MAIT cell absolute number
Fold change over unchallenged control
Naïve mice
WT
IL18R -/-
IFNaR -/-
E
Day 3 MAIT cells as % αβ T cells
Fold change over unchallenged control
Naïve mice
F
Day 3 CD69+ as % MAIT cells
Fold change over unchallenged control
Naïve mice
G
Absolute MAIT cell numbers
Naïve mice
H
MAIT cells as % αβ T cells
Naïve mice
I
CD69+ as % MAIT cells
Naïve mice
J
Absolute Non-MAIT T cell numbers
Naïve mice
K
Non-MAIT T cell as % αβ T cells
Naïve mice
L
CD69+ as % Non-MAIT T cells
Naïve mice
M
Absolute MAIT cell numbers
Mice pre-infected with Salmonella BRD509
N
MAIT cells as % αβ T cells
Mice pre-infected with Salmonella BRD509
O
CD69+ as % MAIT cells
Mice pre-infected with Salmonella BRD509
P
Absolute Non-MAIT T cell numbers
Mice pre-infected with Salmonella BRD509
Q
Non-MAIT T cells as % αβ T cells
Mice pre-infected with Salmonella BRD509
R
CD69+ as % Non-MAIT T cells
Mice pre-infected with Salmonella BRD509
S
Absolute MAIT cell numbers
Naïve mice, WT vs. IFNαR-/-
MAIT cell absolute number
T
MAIT cell as % αβ T cells
Naïve mice, WT vs. IFNαR-/-
MAIT cells as % of αβ T cells
U
CD69+ as % MAIT cell
Naïve mice, WT vs. IFNαR-/-
CD69+ as % MAIT cells
D0
D3
V
Absolute MAIT cell numbers
Naïve mice, WT vs. IL18R-/-
W
MAIT cell as % αβ T cells
Naïve mice, WT vs. IL18R-/-
X
CD69+ as % MAIT cell
Naïve mice, WT vs. IL18R-/-
WT
IFNαR-/-
IL18R-/-

Fig. S2. Cytokine-modulated accumulation and activation of pulmonary MAIT cells upon bleomycin stimulation; Related to Fig. 1. (A to **C,** and **M** to **R)** Experiments were conducted using mice that had undergone pre-infection of *Salmonella* Typhimurium BRD509 to enrich the MAIT cell population. (**D** to **L**, and **S** to **X**) Experiments were conducted using naïve mice that had not undergone pre-infection of *Salmonella* Typhimurium BRD509. Accumulation and activation of lung MAIT cells in WT C57BL/6 mice or mice with IL18R or IFNαR deficiency following a bleomycin challenge. (**A**) Fold change in the absolute MAIT cell and non-MAIT αβ T cell number in the lungs of **MAIT cell-boosted mice** (infected with *Salmonella* Typhimurium BRD509 four weeks prior to bleomycin challenge) post-bleomycin challenge, relative to unchallenged D0 controls (PBS). Comparisons between MAIT cell subsets and non-MAIT αβ T cell subsets at individual time-points, were conducted using unpaired t tests or Mann-Whitney tests, *$P < 0.05$, **$P < 0.01$, ***$P < 0.001$, ****$P < 0.0001$. (**B**) Fold change in MAIT cell and non-MAIT αβ T cell frequency as the percentage of total pulmonary αβ T cells in the lungs of **MAIT cell-boosted mice** post-bleomycin challenge, relative to unchallenged controls. Comparisons between MAIT cell subsets and non-MAIT αβ T cell subsets at individual time-points, were conducted using unpaired t tests or Mann-Whitney tests, *$P < 0.05$, **$P < 0.01$, ***$P < 0.001$, ****$P < 0.0001$. (**C**) Proportion of pulmonary MAIT cells and non-MAIT αβ T cells expressing CD69. Statistical comparisons across different timepoints post-bleomycin challenge and unchallenged PBS control were made using one-way ANOVA with Dunnett's or Dunn's multiple comparison tests, ▲$P < 0.05$, ▲▲$P < 0.01$, ▲▲▲$P < 0.001$, ▲▲▲▲$P < 0.0001$. Comparisons between MAIT cell subsets and non-MAIT αβ T cell subsets at individual time-points, were conducted using unpaired t tests or Mann-Whitney tests. The data are presented as the mean ± SEM of a single experiment, with 3-5 mice in each group. (**D**) Fold change in the number of pulmonary MAIT cells in naïve WT or cytokine knock out mice on day 3 post-bleomycin challenge, relative to unchallenged controls. (**E**) Fold change in

the frequency of MAIT cells as a percentage of total pulmonary αβ T cells in naïve WT or cytokine knock out mice on day 3 post challenge, relative to unchallenged controls. (**F**) Fold change in the proportion of pulmonary MAIT cells expressing CD69 in naïve WT or cytokine knock out mice on day 3 post challenge, relative to unchallenged controls. For Fig. S2 D to F, differences in significance between WT mice and cytokine deficient mice were assessed using one-way ANOVA with Dunnett's multiple comparison tests. Data are mean ± SEM, pooled from three independent experiments, with 2–5 mice per group in each replicate; $^{*}P < 0.05$, $^{**}P < 0.01$, $^{***}P < 0.001$, $^{****}P < 0.0001$. (**G** to **L**) Raw absolute number data for **Fig.1**, **A to C**. Statistical comparisons across different timepoints post-bleomycin challenge and unchallenged PBS control were made using one-way ANOVA with Dunnett's or Dunn's multiple comparison tests, $^{*}P < 0.05$, $^{**}P < 0.01$, $^{***}P < 0.001$, $^{****}P < 0.0001$. (**M** to **R**) Raw absolute number data for **Fig. S1, A to C**. Statistical comparisons across different timepoints post-bleomycin challenge and unchallenged PBS control were made using one-way ANOVA with Dunnett's or Dunn's multiple comparison tests, $^{*}P < 0.05$, $^{**}P < 0.01$, $^{***}P < 0.001$, $^{****}P < 0.0001$. (**S** to **X**) Raw absolute number data for **Fig. S1, D to F**. Statistical significance tested by two-way ANOVA with Holm-Sidak's multiple comparisons test; $^{*}P < 0.05$, $^{**}P < 0.01$, $^{***}P < 0.001$, $^{****}P < 0.0001$.

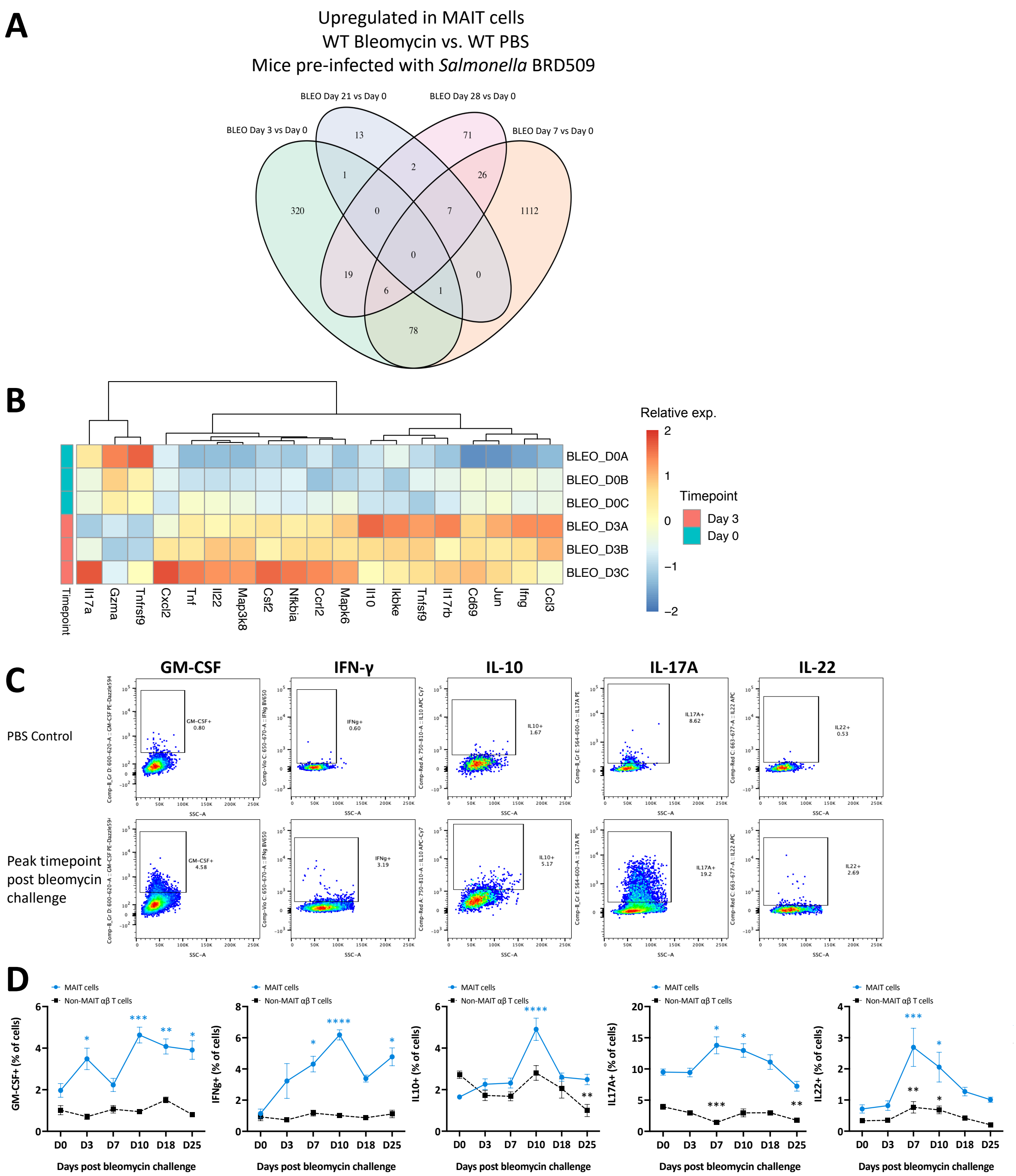

Fig. S3: Gene expression and cytokine profile of pulmonary MAIT cells following bleomycin-induced lung injury in MAIT cell-boosted mice; Related to Fig. 1. (**A** to **D**) Experiments were conducted using mice that had undergone pre-infection of *Salmonella* Typhimurium BRD509 to enrich the MAIT cell population. (**A**) Venn diagram showing the overlap of genes up regulated in mice lung MAIT cells on day 3, 7, 21 and 28 post-bleomycin challenge, compared to unchallenged controls. (**B**) Heatmap of cytokines/chemokines

expression in lung MAIT cells at D0 and D3 post bleomycin. (**C**) Flow cytometry plot illustrating the expression levels of GM-CSF, IFN-γ, IL-10, IL-17A, and IL-22 in lung MAIT cells from MAIT-cell enriched mice after bleomycin challenge. Peak response times to bleomycin for each cytokine are presented, with Day 10 for GM-CSF, IFN-γ, and IL-10, and Day 7 for IL-17A and IL-22. (**D**) Proportion of pulmonary MAIT cells expressing selected cytokines as percentage of MAIT and non-MAIT αβ T cells. Pooled mean ± SEM from two independent experiments with similar results, with 3–5 mice per group in each replicate. Statistical tests compare each time-point with day 0 control in each subset by one-way ANOVA with Dunnett's or Kruskal-Wallis with Dunn's *versus* D0. *P<0.05, **P <0.01, ***P<0.001, ****P<0.0001.

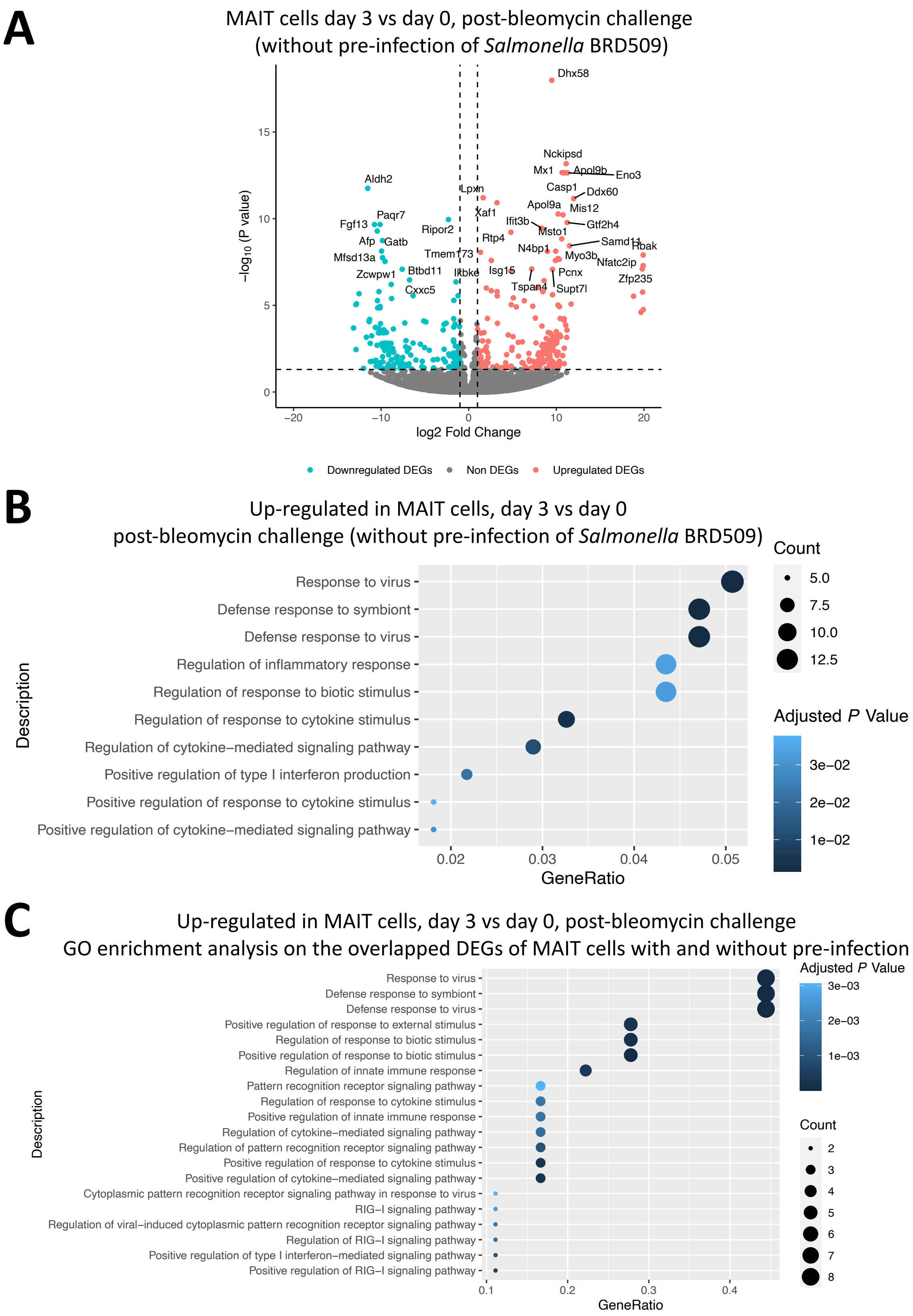

Fig. S4. Gene expression profile of pulmonary MAIT cells following bleomycin challenge in naïve mice; Related to Fig. 1. (**A** to **C**) Experiments were conducted using naïve mice that

had not undergone pre-infection of *Salmonella* Typhimurium BRD509. (**A**) Volcano plot of differentially expressed genes (DEGs) [log2 fold change (FC) > 1, adjusted $P < 0.05$] in mice lung MAIT cells on day 3 post-bleomycin challenge compared to PBS controls (n = 3 per group). The top up and down-regulated genes are annotated. Horizontal line indicates P value threshold of 0.05. Vertical line indicates log2 fold change threshold of 1. (**B**) All significantly enriched ($P < 0.05$) pathways from Gene Ontology (GO) database (biological process) in upregulated DEGs of mice lung MAIT cells at day 3 post-bleomycin challenge compared to PBS controls. (**C**) All significantly enriched ($P < 0.05$) pathways from GO database (biological process) in overlapped DEGs of MAIT cells on day 3 post-bleomycin challenge, with and without pre-infection.

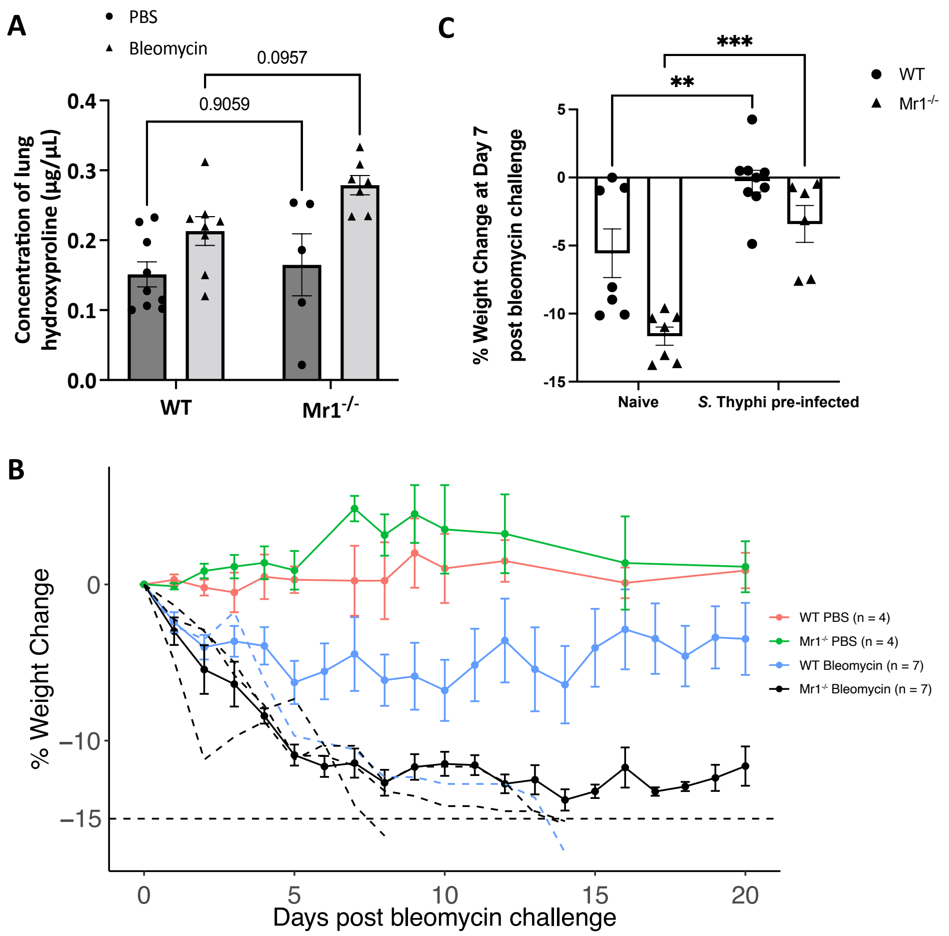

Fig. S5. Differential responses in WT and *Mr1*$^{-/-}$ mice to bleomycin challenge; Related to Fig. 2. (**A**) Hydroxyproline concentration in WT and *Mr1*$^{-/-}$ mice at D21 after bleomycin. Experiments were conducted using mice that had undergone pre-infection of *Salmonella* Typhimurium BRD509 to enrich the MAIT cell population. Data are one representative experiment of two independent experiments, with 5–9 mice per group in each replicate. Statistical significance tested by two-way ANOVA with Holm-Sidak's multiple comparisons test. (**B**) Body weight loss expressed as a percentage of the weight before bleomycin challenge. Experiments were conducted using naïve mice that had not undergone pre-infection with *Salmonella* Typhimurium BRD509. Data are one representative experiment of two independent experiments, with 4–7 mice per group in each replicate. Graphs show mean weights ± SEM for all mice within the group, with individual plots (dotted line) for those animals that succumbed to the bleomycin challenge. (**C**) Body weight loss of naïve and *Salmonella* Typhimurium BRD509 pre-infected mice at day 7 post-bleomycin challenge, expressed as a percentage of the baseline weight prior to the challenge. Bleomycin dose: 1.0 U/kg (body weight) for naïve mice, 1.875 U/kg for *Salmonella* Typhimurium BRD509 pre-infected mice. Data (mean ± SEM) are combined from two independent experiments, with 3–5 mice per group in each replicate. Significance was tested by two-way ANOVA with Sidak's multiple comparisons test.

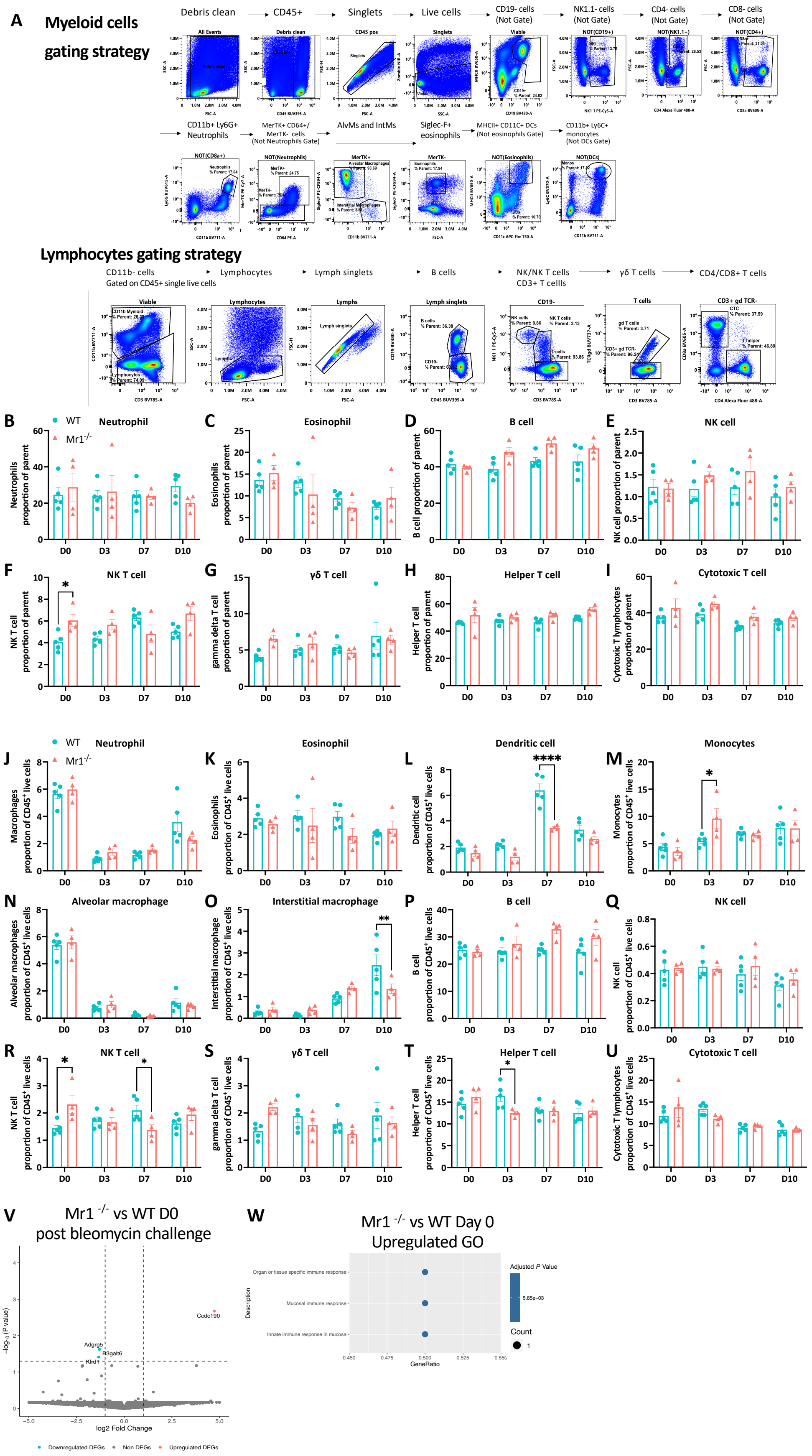
A
Myeloid cells gating strategy
Debris clean
CD45+
Singlets
Live cells
CD19- cells (Not Gate)
NK1.1- cells (Not Gate)
CD4- cells (Not Gate)
CD8- cells (Not Gate)
CD11b+ Ly6G+ Neutrophils
MerTK+ CD64+/ MerTK- cells (Not Neutrophils Gate)
AlvMs and IntMs
Siglec-F+ eosinophils
MHCII+ CD11C+ DCs (Not eosinophils Gate)
CD11b+ Ly6C+ monocytes (Not DCs Gate)
Lymphocytes gating strategy
CD11b- cells Gated on CD45+ single live cells
Lymphocytes
Lymph singlets
B cells
NK/NK T cells CD3+ T cells
γδ T cells
CD4/CD8+ T cells
B
WT
Mr1-/-
Neutrophil
Neutrophils proportion of parent
C
Eosinophil
Eosinophils proportion of parent
D
B cell
B cell proportion of parent
E
NK cell
NK cell proportion of parent
F
NK T cell
NK T cell proportion of parent
G
γδ T cell
gamma delta T cell proportion of parent
H
Helper T cell
Helper T cell proportion of parent
I
Cytotoxic T cell
Cytotoxic T lymphocytes proportion of parent
D0
D3
D7
D10
J
Neutrophil
Macrophages proportion of CD45+ live cells
K
Eosinophil
Eosinophils proportion of CD45+ live cells
L
Dendritic cell
Dendritic cell proportion of CD45+ live cells
M
Monocytes
Monocytes proportion of CD45+ live cells
N
Alveolar macrophage
Alveolar macrophages proportion of CD45+ live cells
O
Interstitial macrophage
Interstitial macrophage proportion of CD45+ live cells
P
B cell
B cell proportion of CD45+ live cells
Q
NK cell
NK cell proportion of CD45+ live cells
R
NK T cell
NK T cell proportion of CD45+ live cells
S
γδ T cell
gamma delta T cell proportion of CD45+ live cells
T
Helper T cell
Helper T cell proportion of CD45+ live cells
U
Cytotoxic T cell
Cytotoxic T lymphocytes proportion of CD45+ live cells
V
Mr1 -/- vs WT D0 post bleomycin challenge
Ccdc190
Adgrg5
B3galt6
Klrd1
-log10 (P value)
log2 Fold Change
Downregulated DEGs
Non DEGs
Upregulated DEGs
W
Mr1 -/- vs WT Day 0 Upregulated GO
Organ or tissue specific immune response
Mucosal immune response
Innate immune response in mucosa
Description
GeneRatio
Adjusted P Value
5.85e-03
Count

Fig. S6. Pulmonary cellular dynamics and gene expression in both WT and *Mr1*$^{-/-}$ mice following bleomycin challenge; Related to Fig. 2. (**A** to **W**) Experiments were conducted using mice that had undergone pre-infection of *Salmonella* Typhimurium BRD509 to enrich the MAIT cell population. (**A**) Flow cytometry gating strategy for the identification of pulmonary myeloid cells and lymphocytes. Representative flow cytometry plots showing gating for cells isolated from mouse lung tissue to identify neutrophil (CD11b^{+} Ly6G^{+}), eosinophil (MerTK^{-} Siglec-F^{+}), alveolar macrophage (MerTK^{+} CD64^{+} CD11b^{-} Siglec-F^{+}), interstitial macrophage (MerTK^{+} CD64^{+} CD11b^{+} SiglecF^{-}), dendritic cell (MerTK^{-} CD11c^{+} MHCII^{+}), monocyte (MerTK^{-} CD64^{+} CD11b^{+} Ly6C^{+}), B cell (CD3- CD19+), CD4^{+} T cell (CD3^{+} CD19^{-} CD4^{+}), CD8^{+} T cell (CD3^{+} CD19^{-} CD8^{+}), NK cells (CD3^{-} NK1.1^{+}), NK T cell (CD3^{+} NK1.1^{+}), and γδ-T cell (TCRγδ^{+}) populations. (**B** to **I**) Frequencies of neutrophil (**B**), eosinophil (**C**), B cell (**D**), NK cell (**E**), NK T cell (**F**), γδ-T cell (**G**), helper T cell (**H**) and cytotoxic T cell (**I**) **as percentages of parent** in WT and *Mr1*$^{-/-}$ mice lungs. (**J** to **U**) Frequencies of neutrophil (**J**), eosinophil (**K**), dendritic cell (**L**), monocyte (**M**), alveolar macrophage (**N**), interstitial macrophage (**O**), B cell (**P**), NK cell (**Q**), NK T cell (**R**), γδ-T cell (**S**), helper T cell (**T**) and cytotoxic T cell (**U**) **as percentages of total live immune cells** (Live CD45^{+} cells) in WT and *Mr1*$^{-/-}$ mice lungs. Data are one representative experiment of two independent experiments, with 4–6 mice per group in each replicate. Graphs show mean ± SEM. Statistical significance tested by two-way ANOVA with Holm-Sidak's multiple comparisons test; **P* < 0.05. (**V**) Volcano plot of DEGs [log2 fold change (FC) > 1, adjusted *P* < 0.05] in whole lung tissue from WT and *Mr1*$^{-/-}$ mice lungs without bleomycin challenge (Day 0). The top 25 up and down-regulated genes are labelled. Horizontal line indicates *P* value threshold of 0.05. Vertical line indicates log2 fold change threshold of 1. (**W**) All significantly enriched (*P* < 0.05) upregulated pathways from Gene Ontology (GO) database (biological process) in upregulated DEGs in the lungs of *Mr1*$^{-/-}$ mice compared with WT mice lungs

without bleomycin challenge (Day 0). Colour intensity indicates the statistical significance of the upregulation, and dot size signifies the number of genes upregulated in the pathway. The x axis denotes the proportion of all DEGs included in the pathway (Gene Ratio).

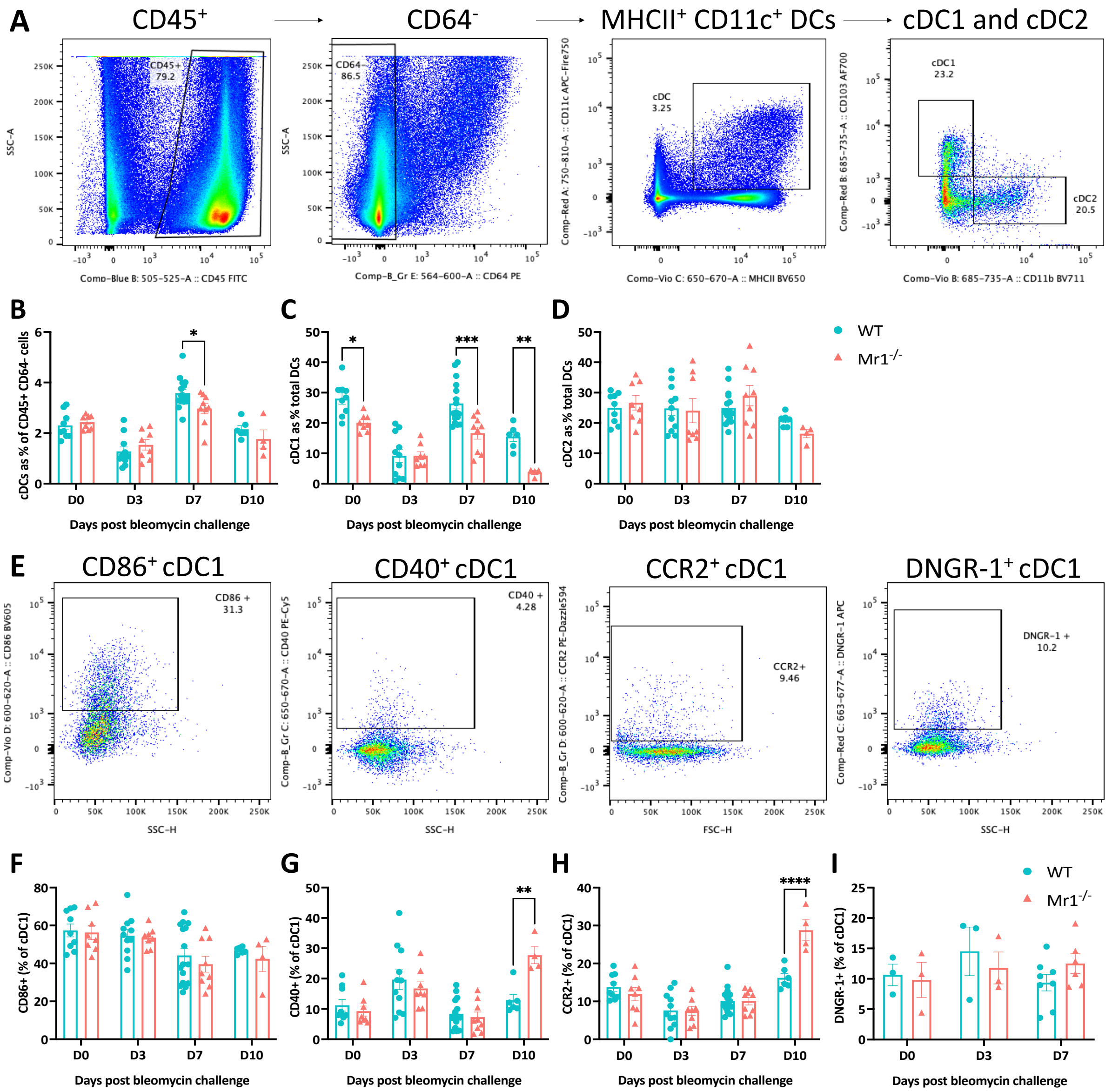

Fig. S7. Characterisation and activation status of mouse lung conventional dendritic cells subsets; Related to Fig. 3. (**A**) Representative flow cytometry plots of lung conventional dendritic cells (cDC), type 1 cDCs (cDC1) and type 2 cDCs (cDC2). (**B**) Frequencies of cDC as percentages of total CD45^{+} CD64^{-} cells in WT and *Mr1*$^{-/-}$ mice lungs. (**C** and **D**) Frequencies of cDC1 (**C**) and cDC2 (**D**) as percentages of total cDC population in WT and *Mr1*$^{-/-}$ mice

lungs. Data represent combined data from two (Day 0, 3 and 7) or one (Day 10) independent experiments, with 3-6 mice per group. (**E**) Representative flow cytometry plots showing expression of CD86, CD40, CCR2 and DNGR-1, gated on live pulmonary $CD45^{+}$ $CD64^{-}$ $CD11c^{+}$ $MHCII^{+}$ $CD103^{+}$ cDC1 cells. (**F** to **I**) Fraction of pulmonary cDC1 cells expressing CD86 (**F**), CD40 (**G**), CCR2 (**H**) and DNGR-1 (**I**) expressed as a percentage of pulmonary cDC1 in lungs of WT and $Mr1^{-/-}$ mice. For CD86, CD40 and CCR2 expression on cDC1 cells, data represent combined data from two (Day 0, 3 and 7) or one (Day 10) independent experiments, with 3-6 mice per group. For DNGR-1 expression on cDC1 cells, data represent combined data from two (Day 7) or one (Day 0 and 3) independent experiments, with 2-4 mice per group. Data are presented as mean ± SEM. Significance was tested by two-way ANOVA with Sidak's multiple comparisons test; $*P < 0.05$, $**P < 0.01$, $***P < 0.001$, $****P < 0.0001$.

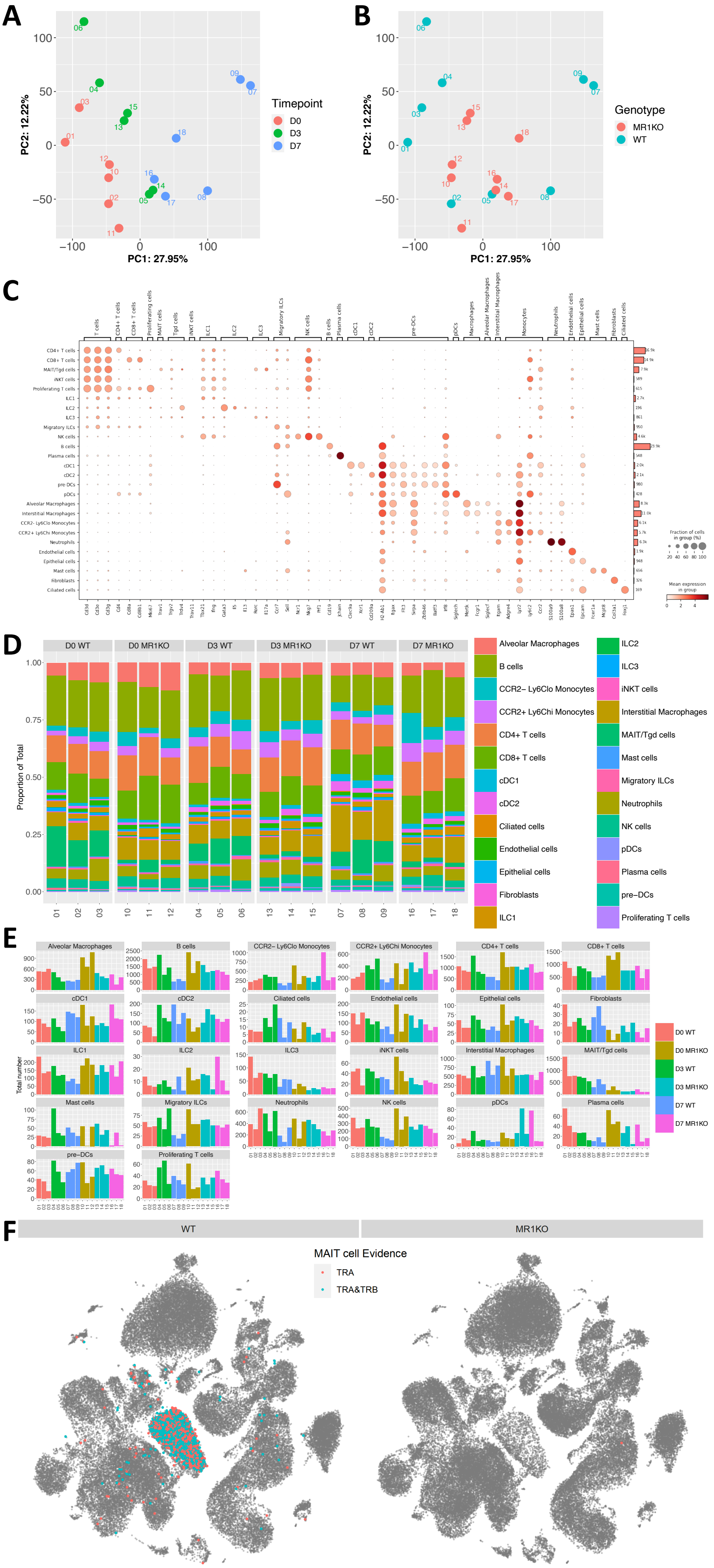
A
PC2: 12.22%
PC1: 27.95%
Timepoint
D0
D3
D7
B
PC2: 12.22%
PC1: 27.95%
Genotype
MR1KO
WT
C
Fraction of cells in group (%)
Mean expression in group
D
D0 WT
D0 MR1KO
D3 WT
D3 MR1KO
D7 WT
D7 MR1KO
Proportion of Total
Alveolar Macrophages
B cells
CCR2− Ly6Clo Monocytes
CCR2+ Ly6Chi Monocytes
CD4+ T cells
CD8+ T cells
cDC1
cDC2
Ciliated cells
Endothelial cells
Epithelial cells
Fibroblasts
ILC1
ILC2
ILC3
iNKT cells
Interstitial Macrophages
MAIT/Tgd cells
Mast cells
Migratory ILCs
Neutrophils
NK cells
pDCs
Plasma cells
pre−DCs
Proliferating T cells
E
Total number
D0 WT
D0 MR1KO
D3 WT
D3 MR1KO
D7 WT
D7 MR1KO
F
WT
MR1KO
MAIT cell Evidence
TRA
TRA&TRB

Fig. S8. Differential gene expression and cellular distribution in mouse lung cell lineages across genotypes and timepoints post-challenge; Related to Fig. 3. (**A** and **B**) Principal component analysis on the overall transcriptomes of each sample coloured by timepoint (**A**) or mice genotype (**B**). (**C**) Dot plot showing the top discriminative gene sets for each cell lineage compared with every other lineage. Dot size represents fraction of cells in each group. Colour scales denote the normalised mean gene expression for each cluster. Bar chart on the right represent number of cells of each cell type in the single-cell RNA sequencing dataset. (**D** and **E**) Proportion distribution (**D**) and absolute number (**E**) of the 27 cell lineages per sample. Bars represent the absolute number (**D**) or percentage (**E**) of cells assigned to each colour-coded cell lineage relative to the total cells for each sample. (**F**) UMAP illustrating the presence of MAIT cells in both WT and *Mr1*$^{-/-}$ mice. MAIT cells were identified using TCR sequencing results generated by Cell Ranger. A cell is classified as a MAIT cell if it belongs to a clonotype with expression of *Trav1* and *Traj33* (TRA), or a clonotype with the expression of *Trav1* and *Traj33*, paired with either *Trbv19* or *Trbv13* (TRA & TRB) (https://support.10xgenomics.com/single-cell-vdj/software/pipelines/latest/algorithms/inkt-mait).

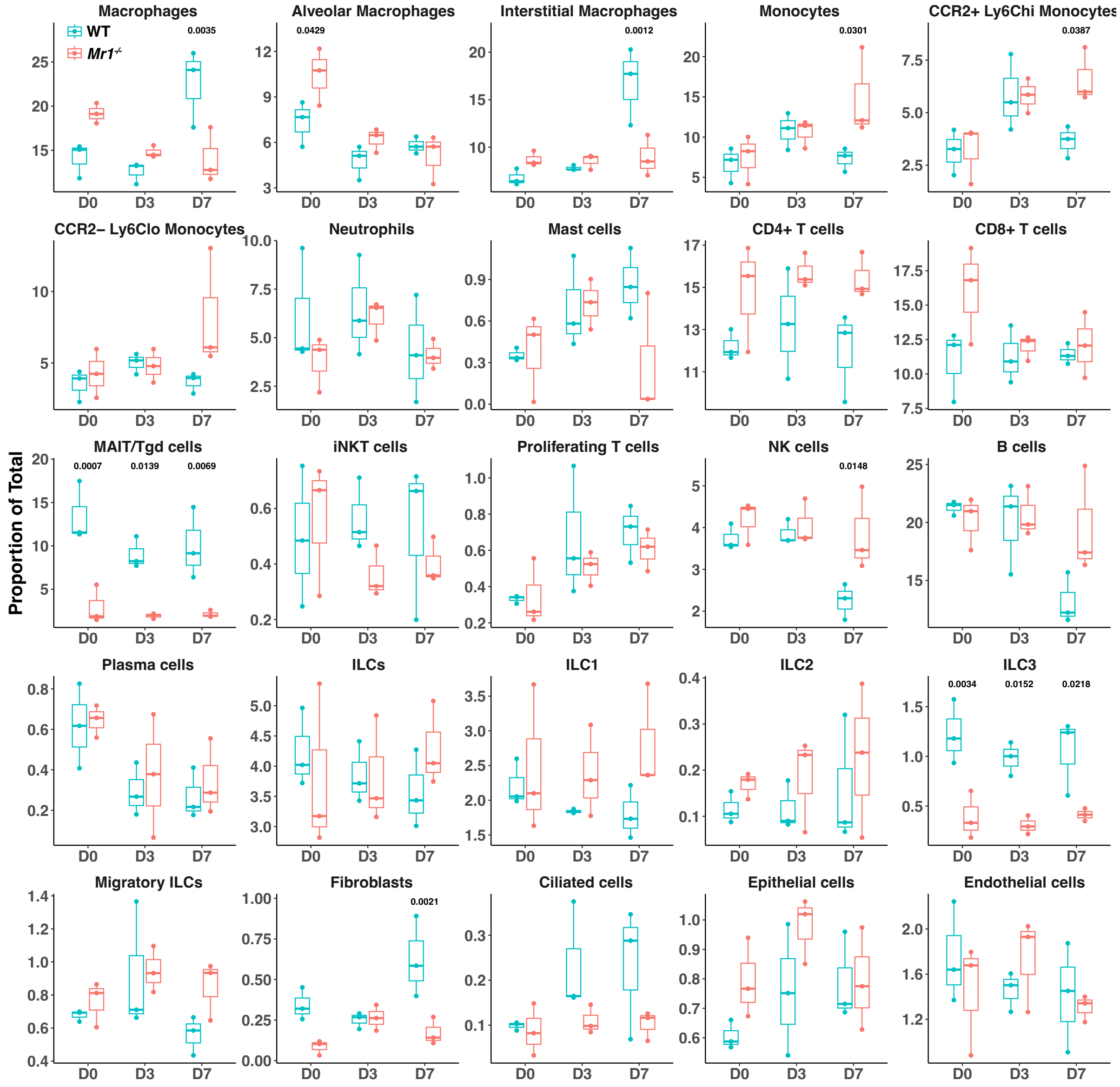

Fig. S9. Cell type distribution in both WT and *Mr1*$^{-/-}$ mouse lungs post-bleomycin challenge; Related to Fig. 3. Proportion of the indicated cell types of total lung cells was calculated for individual mice at the indicated time points after bleomycin challenge and for PBS treated control mice (n = 3 for each genotype). *P* values generated using a two-way ANOVA with Sidak's multiple comparisons test.

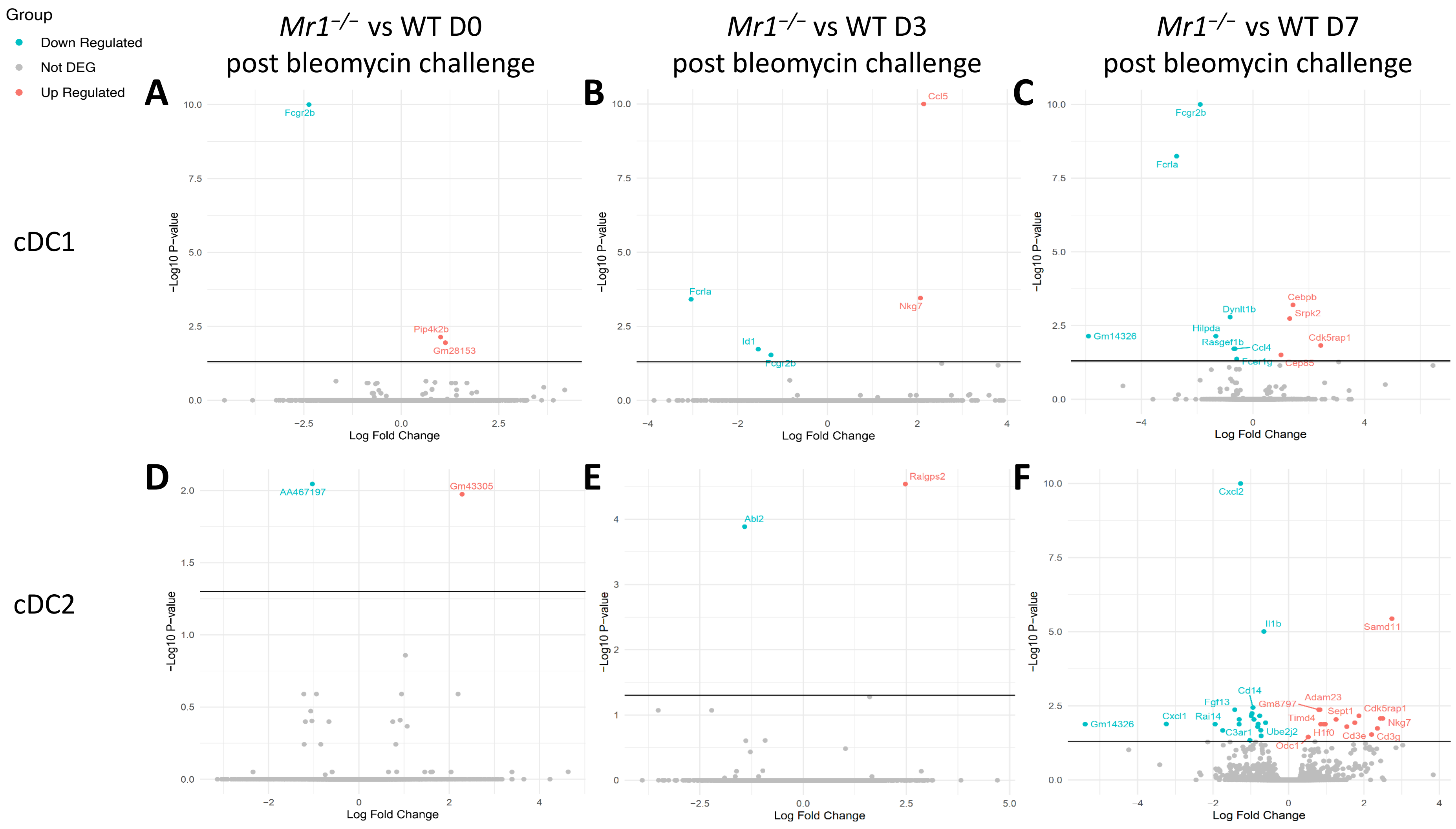

Group
Down Regulated
Not DEG
Up Regulated
Mr1−/− vs WT D0 post bleomycin challenge
Mr1−/− vs WT D3 post bleomycin challenge
Mr1−/− vs WT D7 post bleomycin challenge
cDC1
cDC2
A
B
C
D
E
F
Fcgr2b
Pip4k2b
Gm28153
Ccl5
Fcrla
Nkg7
Id1
Fcgr2b
Fcrla
Cebpb
Srpk2
Dynlt1b
Gm14326
Hilpda
Cdk5rap1
Rasgef1b
Ccl4
Fcer1g
Cep85
AA467197
Gm43305
Ralgps2
Abl2
Cxcl2
Il1b
Samd11
Cd14
Fgf13
Gm8797
Adam23
Sept1
Cdk5rap1
Timd4
Nkg7
Cxcl1
Rai14
Gm14326
C3ar1
Ube2j2
H1f0
Cd3e
Cd3g
Odc1
−Log10 P−value
Log Fold Change

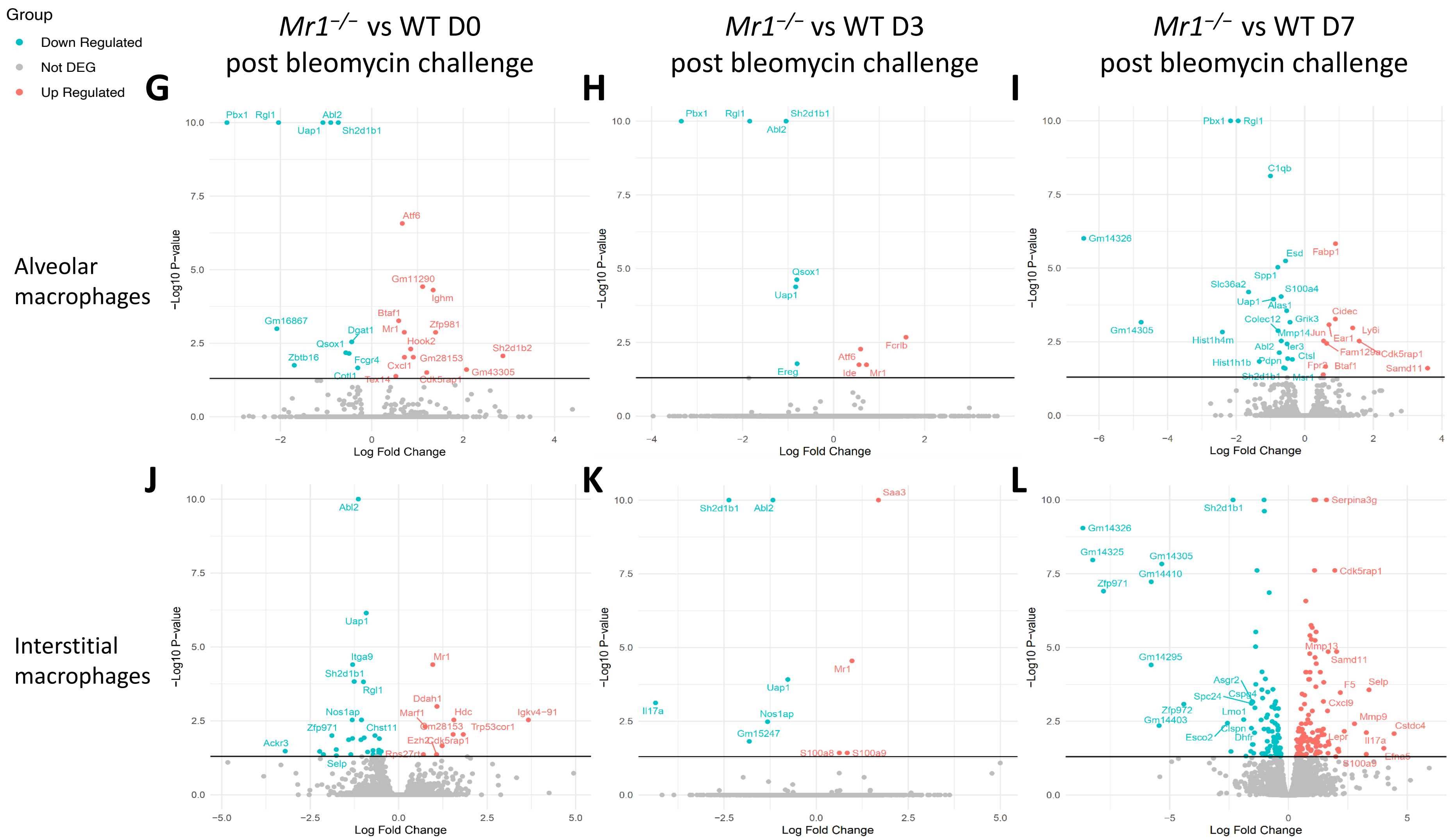

Group
Down Regulated
Not DEG
Up Regulated
Mr1−/− vs WT D0 post bleomycin challenge
Mr1−/− vs WT D3 post bleomycin challenge
Mr1−/− vs WT D7 post bleomycin challenge
Alveolar macrophages
Interstitial macrophages
G
H
I
J
K
L
−Log10 P−value
Log Fold Change
Pbx1
Rgl1
Abl2
Uap1
Sh2d1b1
Atf6
Gm11290
Ighm
Btaf1
Mr1
Zfp981
Gm16867
Dgat1
Qsox1
Hook2
Gm28153
Sh2d1b2
Zbtb16
Fcgr4
Cxcl1
Gm43305
Cotl1
Tex14
Cdk5rap1
Fcrlb
Atf6
Ide
Ereg
C1qb
Gm14326
Esd
Fabp1
Spp1
Slc36a2
S100a4
Alas1
Cidec
Colec12
Grik3
Gm14305
Mmp14
Jun
Ly6i
Hist1h4m
Ear1
Ier3
Fam129a
Ctsl
Hist1h1b
Pdpn
Fpr2
Btaf1
Samd11
Itga9
Ddah1
Nos1ap
Marf1
Hdc
Igkv4−91
Zfp971
Chst11
Trp53cor1
Ackr3
Ezh2
Rps27rt
Selp
Saa3
Il17a
Gm15247
S100a8
S100a9
Serpina3g
Gm14325
Gm14410
Cdk5rap1
Gm14295
Mmp13
Asgr2
Spc24
Cspg4
F5
Cxcl9
Zfp972
Gm14403
Lmo1
Mmp9
Clspn
Cstdc4
Esco2
Dhfr
Lepr
Efna5

Fig. S10. Differential gene expression in dendritic cell and macrophage populations in *Mr1*$^{-/-}$ vs. WT mice in response to bleomycin; Related to Fig. 3. Volcano plots showing cDC1 (**A** to **C**), cDC2 (**D** to **F**), alveolar macrophages (**G** to **H**) and interstitial macrophage (**J** to **L**)-specific DEGs based on adjusted *P* value < 0.05 between *Mr1*$^{-/-}$ and wild-type mice before and post-bleomycin challenge. Horizontal line indicates *P* value threshold of 0.05.

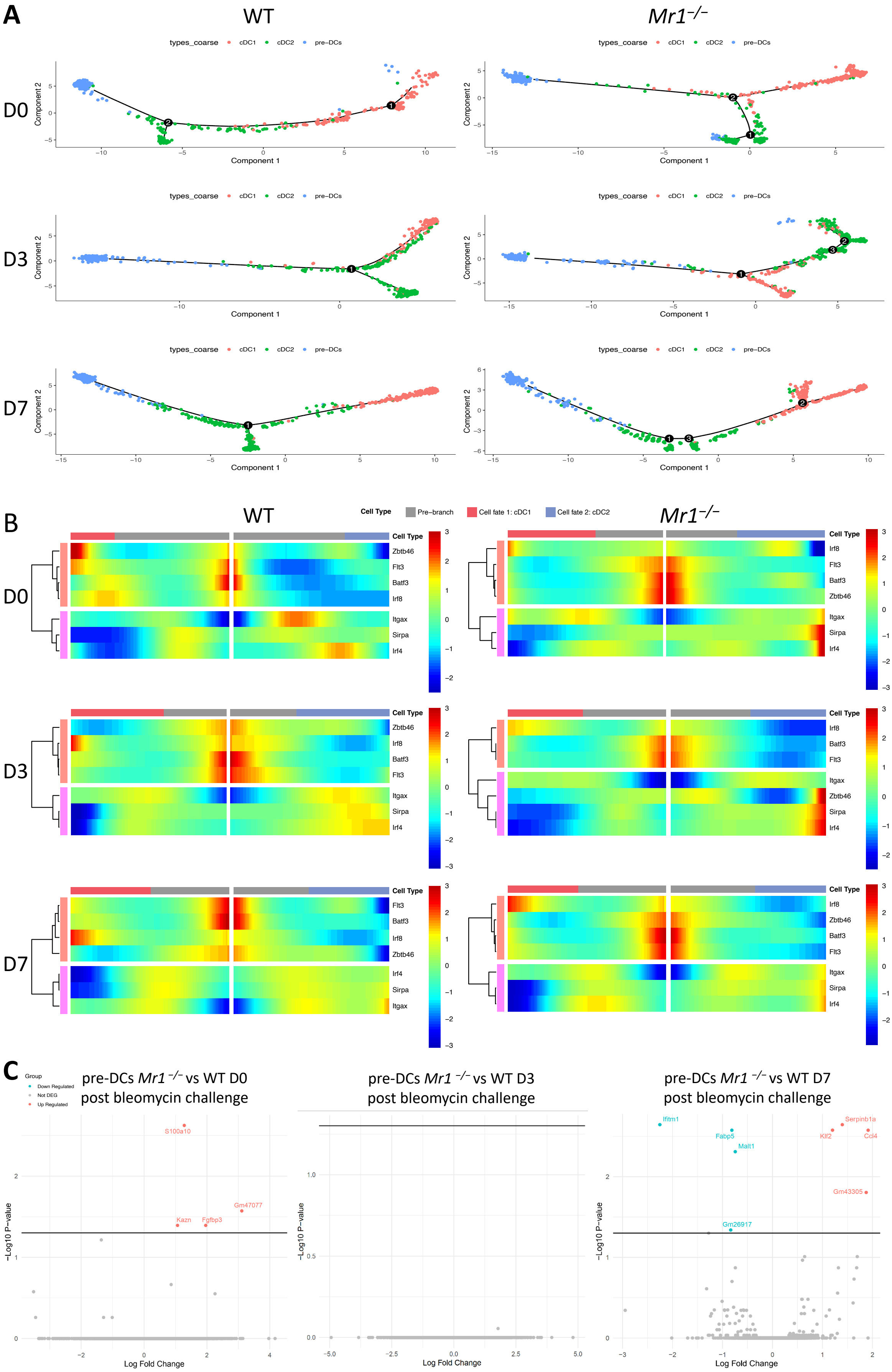
A
WT
Mr1−/−
types_coarse cDC1 cDC2 pre–DCs
D0
D3
D7
Component 1
Component 2
B
WT
Mr1−/−
Cell Type Pre–branch Cell fate 1: cDC1 Cell fate 2: cDC2
Zbtb46
Flt3
Batf3
Irf8
Itgax
Sirpa
Irf4
C
Group
Down Regulated
Not DEG
Up Regulated
pre-DCs Mr1 −/− vs WT D0
post bleomycin challenge
S100a10
Gm47077
Kazn
Fgfbp3
pre-DCs Mr1 −/− vs WT D3
post bleomycin challenge
pre-DCs Mr1 −/− vs WT D7
post bleomycin challenge
Ifitm1
Fabp5
Malt1
Gm26917
Serpinb1a
Klf2
Ccl4
Gm43305
−Log10 P−value
Log Fold Change

Fig. S11. Differentiation trajectories and gene expression patterns of pre-DCs to cDC1 and cDC2 in WT and *Mr1*$^{-/-}$ mouse lungs; Related to Fig. 3. (**A**) Analysis using Monocle2 [S1] showing branches of differentiation trajectory from pre-DCs, the progenitors of cDCs, towards cDC1 and cDC2. (**B**) Heatmap showing dynamic change in gene expression of two differentiation branches (cDC1 and cDC2). Higher gene expression levels are denoted in red, while lower levels in blue. (**C**) Volcano plots showing pre-DC-specific DEGs based on adjusted P value < 0.05 between *Mr1*$^{-/-}$ and WT mice before and post-bleomycin challenge. Horizontal line indicates P value threshold of 0.05.

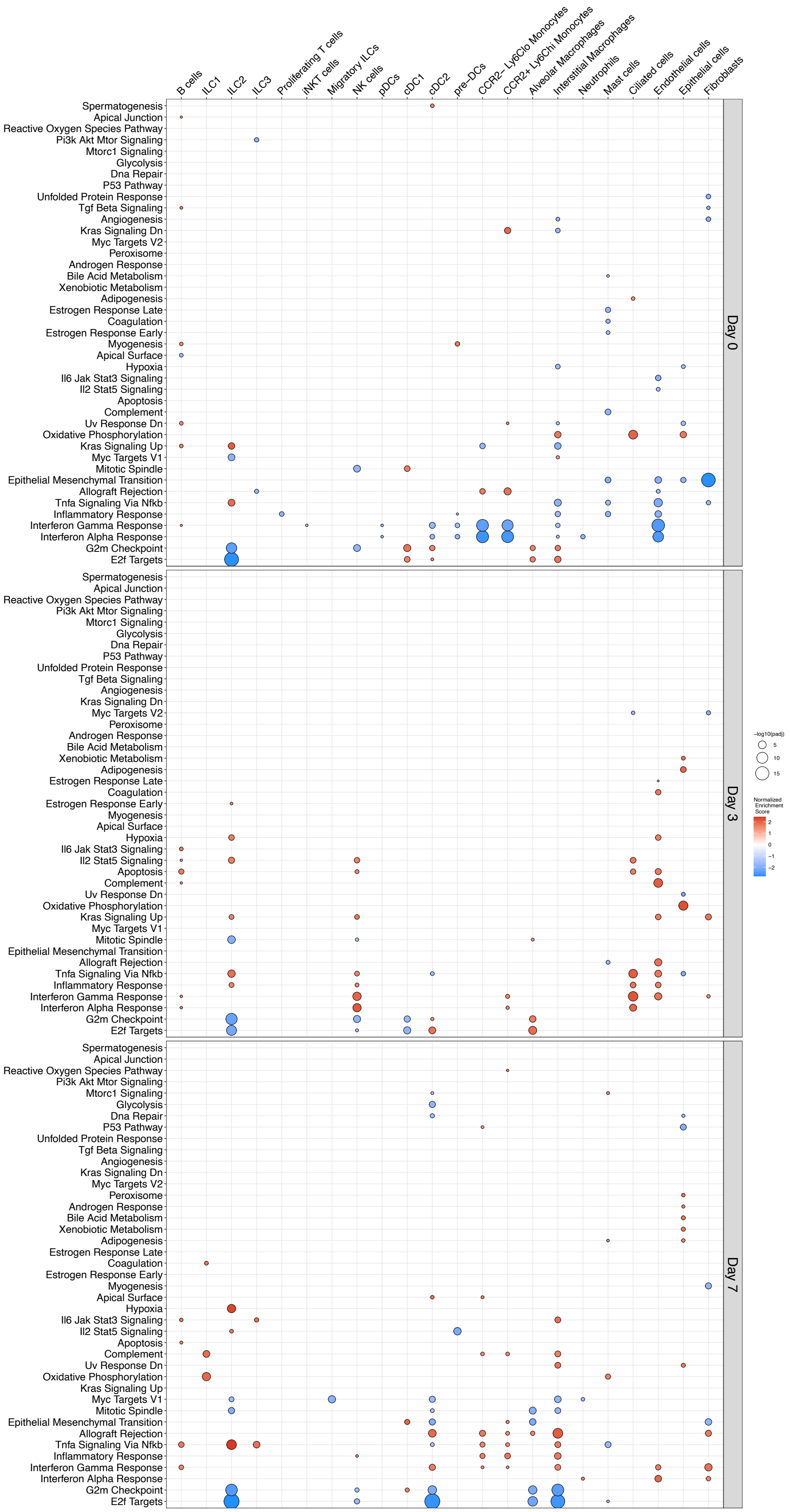

B cells
ILC1
ILC2
ILC3
Proliferating T cells
iNKT cells
Migratory ILCs
NK cells
pDCs
cDC1
cDC2
pre−DCs
CCR2− Ly6Clo Monocytes
CCR2+ Ly6Chi Monocytes
Alveolar Macrophages
Interstitial Macrophages
Neutrophils
Mast cells
Ciliated cells
Endothelial cells
Epithelial cells
Fibroblasts
Spermatogenesis
Apical Junction
Reactive Oxygen Species Pathway
Pi3k Akt Mtor Signaling
Mtorc1 Signaling
Glycolysis
Dna Repair
P53 Pathway
Unfolded Protein Response
Tgf Beta Signaling
Angiogenesis
Kras Signaling Dn
Myc Targets V2
Peroxisome
Androgen Response
Bile Acid Metabolism
Xenobiotic Metabolism
Adipogenesis
Estrogen Response Late
Coagulation
Estrogen Response Early
Myogenesis
Apical Surface
Hypoxia
Il6 Jak Stat3 Signaling
Il2 Stat5 Signaling
Apoptosis
Complement
Uv Response Dn
Oxidative Phosphorylation
Kras Signaling Up
Myc Targets V1
Mitotic Spindle
Epithelial Mesenchymal Transition
Allograft Rejection
Tnfa Signaling Via Nfkb
Inflammatory Response
Interferon Gamma Response
Interferon Alpha Response
G2m Checkpoint
E2f Targets
Day 0
Day 3
Day 7
−log10(padj)
5
10
15
Normalized Enrichment Score
2
1
0
−1
−2

Fig. S12. Gene Set Enrichment Analysis in lung cells of *Mr1*$^{-/-}$ and WT mice post-bleomycin challenge; Related to Fig. 3. Gene Set Enrichment Analysis (GSEA) performed on the *Mr1*$^{-/-}$ and wild-type (WT) mice across different time points utilizing the MSigDB Hallmark collection. Log2 fold changes in differential expression, as computed through DESeq2, served as the rank statistics. The color of the dots represents the Normalised Enrichment Score (NES), while their size indicates the -log10-adjusted *P* value. *P* value was modified using the Benjamini-Hochberg method. The direction of the NES signifies the relative increase or decrease in the *Mr1*$^{-/-}$ mice when compared to the WT mice.

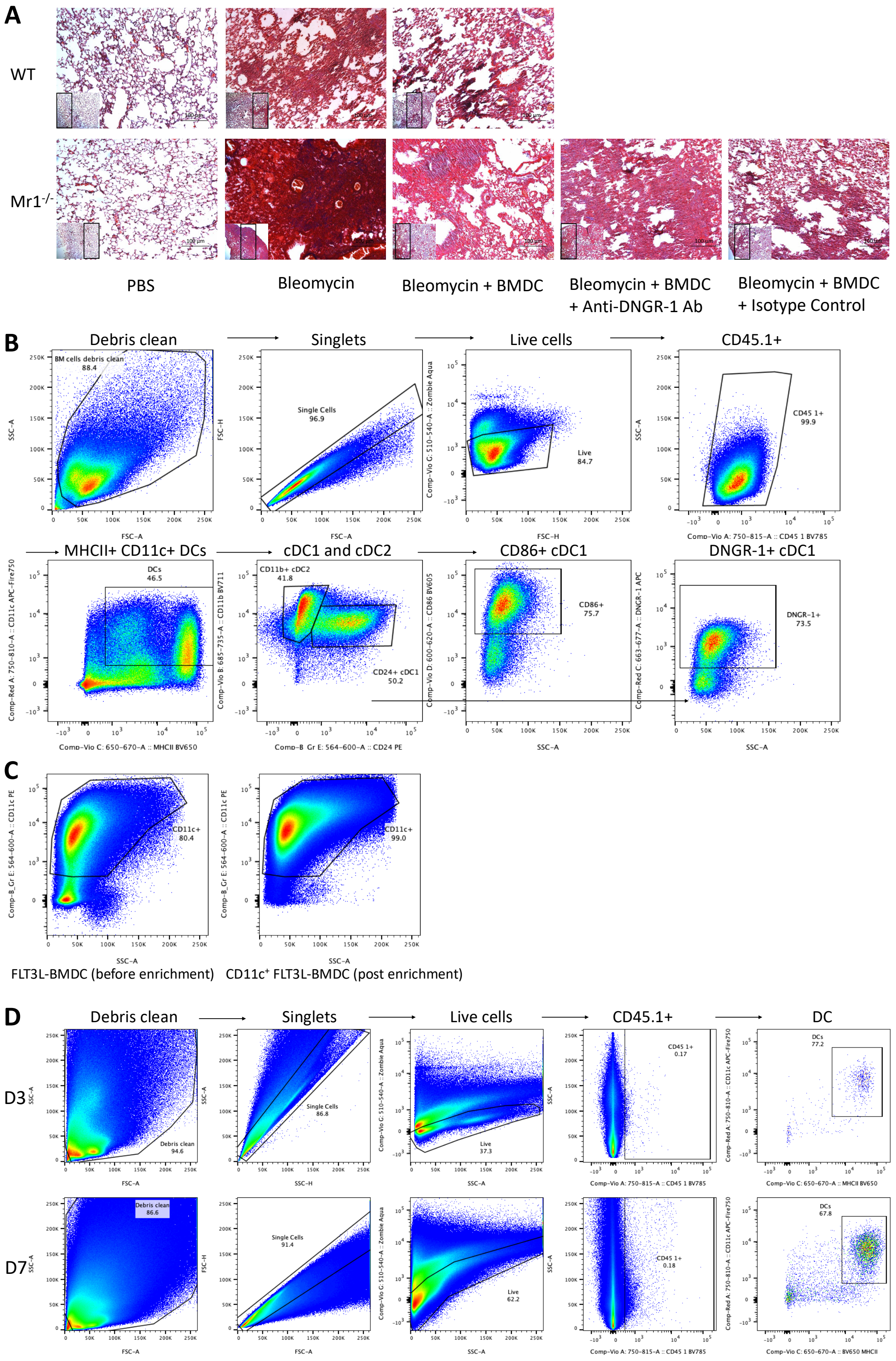

Fig. S13. Lung tissue response and dendritic cell characterisation in WT and *Mr1*$^{-/-}$ mice post-bleomycin challenge and -adoptive transfer of FLT3L-BMDC; Related to Fig. 3. (**A**) Representative images of lung slices of PBS or bleomycin-challenged WT and *Mr1*$^{-/-}$ mice at D21, stained with Masson's trichrome. (**B**) Gating scheme for the identification of conventional

dendritic cells (cDC) in Fms-like tyrosine kinase-3 ligand (Flt3L)-generated bone marrow-derived dendritic cells (FLT3L-BMDC), and expression of CD86 and DNGR-1 on cDC1. cDCs are identified as CD11c^{+} MHCII^{+} cells and can be divided into cDC1 (CD24^{+} CD11b^{-}) and cDC2 (CD24^{-} CD11b^{+}). (**C**) Representative flow plot showing the CD11c^{+} FLT3L-BMDC population before and post-CD11c enrichment (gated on all live CD45.1^{+} cells). (**D**) Flow cytometric analysis of adoptive-transferred CD11c^{+} bone marrow-derived dendritic cells (BMDC, gated as CD45.1^{+} MHCII^{+} CD11c^{+} population) in *Mr1*$^{-/-}$ mice lungs at day 3 and day 7 post-adoptive transfer, respectively.

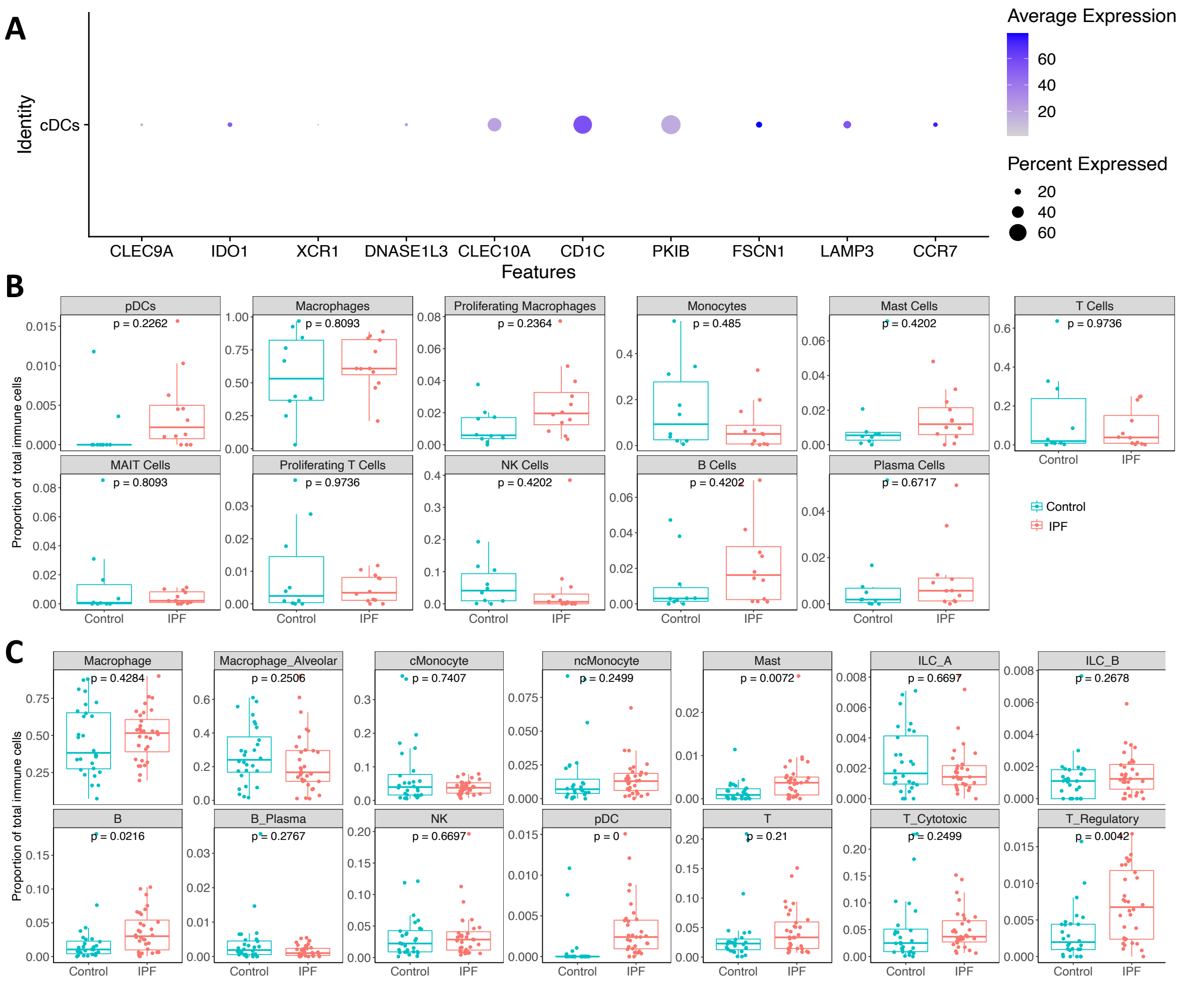

Fig. S14. Gene expression profiling of cDCs in IPF patients' lungs and comparative analysis of cell frequencies between IPF patients and controls; Related to Fig. 4. (**A**) Dot plot showing the gene expression profile for the cDCs in the GSE135893 dataset. Dot size represents percentage of expressed cells. Colour scales denote the average gene expression. (**B** and **C**) Boxplots present frequencies of indicated cell type, as proportions of total lung cells, in IPF patients versus controls, sourced from GSE135893 (**B**) and GSE136831 (**C**). *P* values generated using a two-way ANOVA with Sidak's multiple comparisons test.

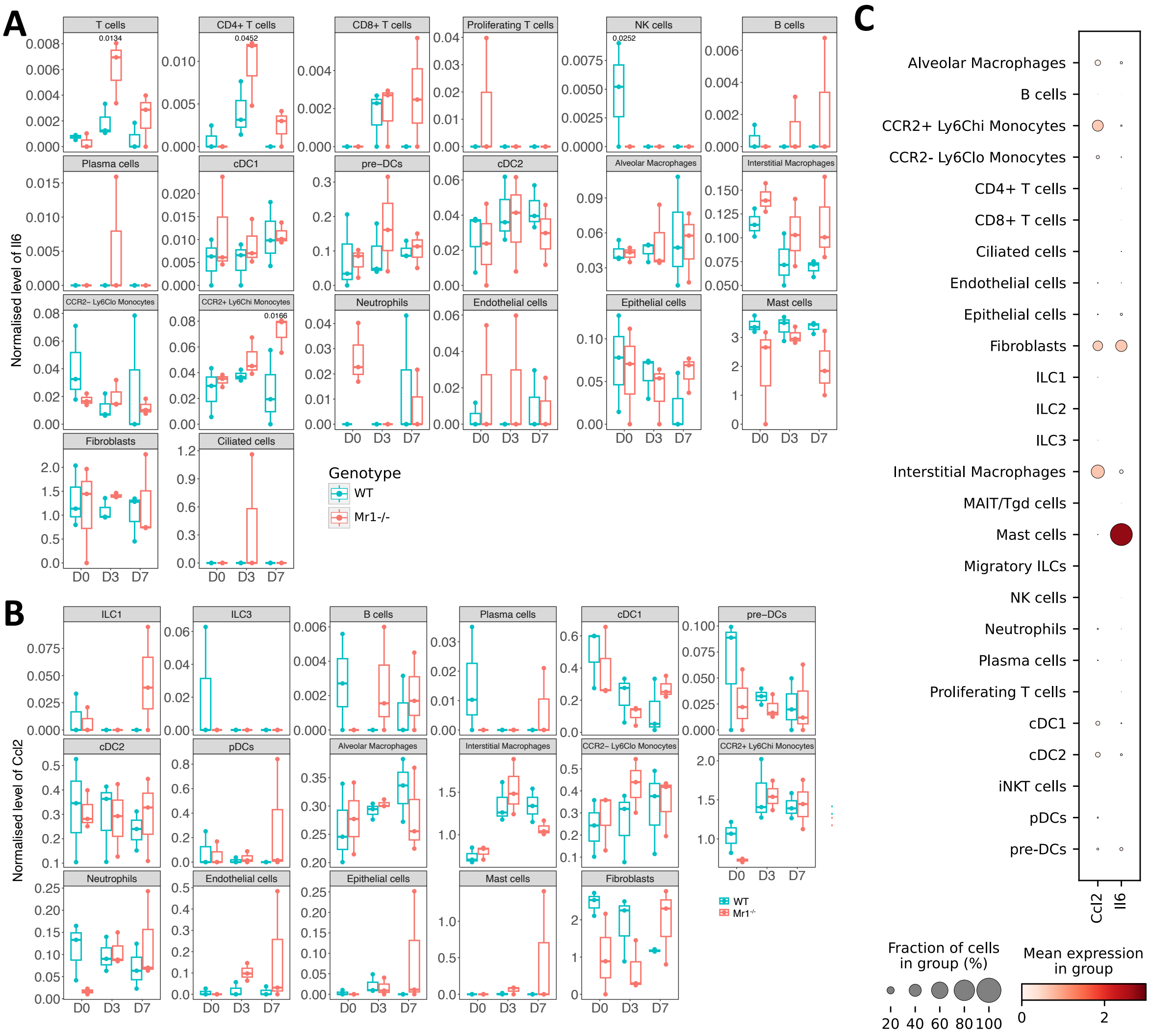

Fig. S15. Differential expression of inflammatory markers Il6 and Ccl2 in lung cell types pre- and post-bleomycin challenge; Related to Fig. 3. Bar plots showing the gene expression

of *Il6* (**A**) and *Ccl2* (**B**) across various cell types pre- and post-bleomycin challenge. (**C**) Dot plot illustrating the combined gene expression levels of *Il6* and *Ccl2* across different cell types, collated from all time points and both WT and *Mr1*$^{-/-}$ mice.

Table S1. DEGs of MAIT cells, IPF vs Control; Related to Fig. 4

	**Log2 Fold Change**	***P* value**	**Adjusted *P* value**
CTD-3252C9.4	3.40847694	5.06E-09	0.00013339
TNFSF9	3.00877575	1.20E-07	0.00316397
CCL4L2	2.67002871	1.65E-10	4.36E-06
ITM2C	2.39843978	2.32E-09	6.13E-05
HSPA1B	2.37900959	1.22E-20	3.21E-16
DUSP4	2.37889768	7.97E-08	0.0021023
CDKN1A	2.20750059	7.27E-07	0.01917007
HSPA1A	2.01768082	5.54E-17	1.46E-12
RGS1	1.95067011	4.28E-16	1.13E-11
PHLDA1	1.91687999	4.00E-10	1.06E-05
CCL3	1.86015604	1.26E-07	0.00332594
NR4A1	1.82189329	2.85E-10	7.51E-06
EGR1	1.78128101	5.63E-08	0.00148433
CSRNP1	1.74935216	9.58E-07	0.02526935
SCGB3A1	1.72801781	3.13E-17	8.25E-13
SCGB1A1	1.69915943	9.92E-14	2.62E-09
SERTAD1	1.69208413	6.91E-07	0.01821557
PDE4B	1.64592784	1.05E-07	0.00275658
BIRC3	1.61577906	1.66E-07	0.00437926
FOSB	1.50301559	4.32E-10	1.14E-05
DNAJB1	1.24869004	8.04E-15	2.12E-10
DNAJA1	1.22687395	3.24E-12	8.53E-08

RGCC	1.21337533	5.22E-09	0.00013778
RPS4Y1	1.20033202	1.36E-08	0.00035806
CORO1B	1.19148334	6.53E-09	0.00017236
BTG2	1.11280058	1.02E-10	2.68E-06
CAPG	1.08636027	9.99E-07	0.02635963
NR4A2	1.07056276	6.34E-07	0.01673196
CCL4	0.98464434	2.16E-07	0.00569644
FOS	0.92669826	2.02E-17	5.32E-13
PPP1R15A	0.89848942	7.88E-07	0.02077662
TNFAIP3	0.89036899	6.43E-10	1.70E-05
PTPRCAP	0.80498537	7.99E-10	2.11E-05
NFKBIA	0.69743629	1.23E-10	3.24E-06
LGALS1	0.64854233	2.07E-07	0.00547243
KLF6	0.64467035	2.59E-11	6.84E-07
ALOX5AP	0.60331068	3.17E-07	0.00837339
ZFP36	0.58343053	1.52E-12	4.01E-08
HLA-DRB1	0.56565865	1.16E-07	0.00305218
DYNLL1	0.56083732	9.13E-07	0.02409233
CD69	0.54163104	3.29E-07	0.00868113
DUSP1	0.52541753	1.67E-10	4.41E-06
JUNB	0.46129731	3.00E-08	0.00079207
HSP90AA1	0.45594655	8.42E-11	2.22E-06
CRIP1	0.42043194	6.61E-07	0.01744826
S100A11	0.41303276	6.73E-07	0.01775344
CD74	0.3775569	5.61E-08	0.00148106

FTH1	0.34485861	1.08E-12	2.84E-08
VIM	0.27986256	1.34E-06	0.03524605
RPS26	-0.4863287	5.11E-15	1.35E-10
PCBP2	-0.6295141	3.05E-16	8.04E-12
PRF1	-0.8782519	3.43E-09	9.05E-05
TRGV10	-1.9454736	2.45E-08	0.00064673
MTRNR2L12	-2.71823	2.10E-07	0.00553425
TRAV12-1	-4.0025993	2.32E-09	6.13E-05

Table S2. Flow cytometry reagents; Related to STAR Methods

No.	Antigen	Conjugation	Clone	Host	Reactivity	Isotype	Concentration Titration	Catalogue number	Supplier
1	CD103	AF700	2 E7	Hamster	Mouse	Armenian Hamster IgG	1:100	121442	BioLegend
2	CD45	BUV395	30-F11	Rat	Mouse	Rat IgG2b, κ	1:100	564279	BD Biosciences
3	CD11c	APC/Fire™ 750	N418	Rat	Mouse	Armenian Hamster IgG	1:100	117352	Biolegend
4	CD19	BV480	1D3	Rat	Mouse	Lewis IgG2a, κ	1:100	566107	BD Biosciences
5	MERTK (Mer)	PE-Cy7	2B10C42	Rat	Mouse	Rat IgG2a, κ	1:100	151522	Biolegend
6	CD4	AF488	GK1.5	Rat	Mouse	Rat IgG2b, κ	1:100	100423	Biolegend
7	Ly-6G	BUV615	1A8	Rat	Mouse	Lewis IgG2a, κ	1:100	751263	BD Biosciences
8	Ly-6C	BV570	HK1.4	Rat	Mouse	Rat IgG2c, κ	1:100	128029	Biolegend
9	MHC II	BV650	M5/114.15.2	Rat	Mouse	Rat IgG2b, κ	1:100	107641	Biolegend
10	CD8a	BV605	53-6.7	Rat	Mouse	Rat IgG2a, κ	1:100	100744	Biolegend
11	CD11b	BV711	M1/70	Rat	Mouse/Human	Rat IgG2b, κ	1:100	101242	Biolegend
12	CD3	BV785	17A2	Rat	Mouse	Rat IgG2b, κ	1:100	100232	Biolegend
13	CD64	PE	X54-5/7.1	Mouse	Mouse	Mouse IgG1, κ	1:100	139304	Biolegend
14	NK1.1	PE/Cyanine5	PK136	Mouse	Mouse	Mouse IgG2a, κ	1:100	108716	Biolegend
15	CD44	BUV805	IM7	Rat	Mouse	Rat IgG2b, κ	1:100	741921	BD Biosciences
16	TCR γ/δ	BUV737	GL3	Hamster	Mouse	Armenian Hamster IgG2, κ	1:100	748991	Biolegend
17	CD19	PerCP Cy5.5	6D5	Rat	Mouse	Rat IgG2a, κ	1:100	115534	BioLegend

18	CD69	FITC	H1.2F3	Hamster	Mouse	Armenian Hamster IgG1, λ3	1:100	553236	BD Biosciences
19	TCRβ	PE-Cy7	H57-597	Hamster	Mouse	Armenian Hamster IgG2, λ1	1:100	560729	BD Biosciences
20	CD25	APC	PC61.5	Rat	Mouse	Rat IgG1, λ	1:100	17-0251-82	eBioscience™
21	CD45.2	BV711	104	Mouse	Mouse	Mouse SJL IgG2a, κ	1:100	563685	BD Biosciences
22	IFN-γ	BV650	XMG1.2	Rat	Mouse	Rat IgG1, κ	1:100	563854	BD Biosciences
23	IL-22	APC	Poly5164	Goat	Mouse	Polyclonal	1:100	516409	BioLegend
24	IL-10	APC-Cy7	JES5-16E3	Rat	Mouse	Rat IgG2b, κ	1:100	505010	BioLegend
25	IL-17A	PE	TC11-18H10.1	Rat	Mouse	Rat IgG1, κ	1:100	506904	BioLegend
26	GM-CSF	PE/Dazzle™ 594	MP1-22E9	Rat	Mouse	Rat IgG2a, κ	1:100	505422	BioLegend
27	SiglecH	PerCPCy5.5	551	Rat	Mouse	Rat IgG1, κ	1:100	129614	BioLegend
28	CD45	FITC	30-F11	Rat	Mouse	Rat IgG2b, κ	1:100	103108	BioLegend
29	CD86	BV605	GL-1	Rat	Mouse	Rat IgG2a, κ	1:100	105037	BioLegend
30	CD40	PE/Cyanine5	44986	Rat	Mouse	Rat IgG2a, κ	1:100	124618	BioLegend
31	CD192	PE/Dazzle™ 594	SA203G11	Rat	Mouse	Rat IgG2b, κ	1:100	150636	BioLegend
32	CD192	APC/Fire™ 750	SA203G11	Rat	Mouse	Rat IgG2b, κ	1:100	150630	BioLegend
33	CD317	PE/Cyanine7	eBio927	Rat	Mouse	Rat IgG2b, κ	1:100	25-3172-82	eBioscience™

34	CD370	APC	7H11	Rat	Mouse	Rat IgG1, κ	1:100	143506	BioLegend
35	CD24	PE/Dazzle™ 594	M1/69	Rat	Mouse	Rat IgG2b, κ	1:100	101838	BioLegend
36	CD45.2	FITC	104	Mouse	Mouse	Mouse (SJL) IgG2a, κ	1:100	109806	BioLegend
37	CD45.1	BV785	A20	Mouse	Mouse	Mouse (A.SW) IgG2a, κ	1:100	110743	BioLegend
38	CD11c	PE	N418	Hamster	Mouse	Armenian Hamster IgG	1:100	117308	BioLegend
39	CD45R	BV421	RA3-6B2	Rat	Mouse	Rat IgG2a, κ	1:100	103251	BioLegend
40	Zombie NIR™ Fixable Viability Kit						1:1000	423106	BioLegend
41	Zombie Aqua™ Fixable Viability Kit						1:1000	423102	BioLegend
42	Zombie Yellow™ Fixable Viability Kit						1:1000	423104	BioLegend

References

S1. Qiu, X., Hill, A., Packer, J., Lin, D., Ma, Y.-A., and Trapnell, C. (2017). Single-cell mRNA quantification and differential analysis with Census. Nat. Methods *14*, 309-315. 10.1038/nmeth.4150.
