# Supplementary material for: MAIT cells protect against sterile lung injury
Source: Cell Rep. Author manuscript; Available in PMC 2025 Jul 17. (PMC7617896; doi:10.1016/j.celrep.2025.115275)
Supplement: Supplementary Materials [file EMS206836-supplement-Supplementary_Materials.docx]

# Supplemental Excel Table Titles and Legends

**Data S1. DEGs of MAIT cells, *Salmonella* Typhimurium BRD509 pre-infected,** **WT B6 bleomycin challenged versus WT B6 unchallenged; Related to Fig. 1**

Differentially expressed genes [log2 fold change (FC) > 1, adjusted *P* < 0.05] of mouse lung MAIT cells on days 3, 7, 14, 21 and 28 post bleomycin challenge (*Salmonella* Typhimurium BRD509 pre-infected), respectively, compared with unchallenged PBS controls.

**Data S2. DEGs of MAIT cells, Naïve, WT B6 bleomycin challenged versus WT B6 unchallenged; Related to Fig. 1**

Differentially expressed genes [log2 fold change (FC) > 1, adjusted *P* < 0.05] of naïve mouse lung MAIT cells on days 3 and 7 post bleomycin challenge, respectively, compared with unchallenged PBS controls.

**Data S3. DEGs of total lung, *Salmonella* Typhimurium BRD509 pre-infected, bleomycin-challenged,** ***Mr1*^−/−^ versus WT B6; Related to Fig. 2**

Differentially expressed genes [log2 fold change (FC) > 1, adjusted *P* < 0.05] in whole lung tissue between *Mr1*^−/−^ and WT mice lungs on days 0, 3, 7, 14 and 21 post bleomycin challenge (*Salmonella* Typhimurium BRD509 pre-infected), respectively.

**Data S4. DEGs, cell type-specific, *Salmonella* Typhimurium BRD509 pre-infected, bleomycin-challenged,** **WT B6 versus *Mr1*^−/−^; Related to Fig. 3**

Differences in cell type-specific differentially expressed genes between WT and *Mr1*^−/−^ mice on days 0 (unchallenged PBS controls), 3 and 7 post bleomycin challenge (*Salmonella* Typhimurium BRD509 pre-infected), respectively.
